# Comparative analysis of COVID-19 guidelines from six countries: a qualitative study on the US, China, South Korea, the UK, Brazil, and Haiti

**DOI:** 10.1186/s12889-020-09924-7

**Published:** 2020-12-03

**Authors:** Ji Youn Yoo, Samia Valeria Ozorio Dutra, Dany Fanfan, Sarah Sniffen, Hao Wang, Jamile Siddiqui, Hyo-Suk Song, Sung Hwan Bang, Dong Eun Kim, Shihoon Kim, Maureen Groer

**Affiliations:** 1 College of Nursing, University of South Florida, 12901 Bruce B. Downs Blvd, Tampa, FL 33612 USA; 2 College of Nursing, University of Tennessee – Knoxville, 1200 Volunteer Blvd, Knoxville, TN 37902 USA; 3 College of Nursing, University of Florida, Health Professions, Nursing, Pharmacy Building, 1225 Center Dr, Gainesville, FL 32603 USA; 4 Morsani College of Medicine, University of South Florida, 12901 Bruce B. Downs Blvd., MDC 78, Tampa, FL 33612 USA; 5 Department of Chemical and Biomedical Engineering, University of South Florida, 4202 E. Fowler Ave, ENB118, Tampa, FL 33620 USA; 6 Canon Medical Systems Ltd., Boundary Court, Gatwick Road, Crawley, RH10 9AX UK; 7 Department of Emergency Medical Service, Daejeon Health Institute of Technology, 21 Chungjeong St., Dong-gu, Daejeon, 34504 Republic of Korea; 8 Department of Special Warfare Medical Non-Commissioned Officer, Daejeon Health Institute of Technology, 21 Chungjeong St., Dong-gu, Daejeon, 34504 Republic of Korea; 9 Department of Disaster Construction Safety, Daejeon Health Institute of Technology, 21 Chungjeong St., Dong-gu, Daejeon, 34504 Republic of Korea; 10 Department of Public Health, Konyang University, 158, Gwasnjeoddong-ro, Seo-gu, Daejeon, 35365 Republic of Korea

**Keywords:** COVID-19, Coronavirus, Government guidelines, Outbreak COVID-19, Pandemic

## Abstract

**Background:**

In late January, a worldwide crisis known as COVID-19 was declared a Public Health Emergency of International Concern by the WHO. Within only a few weeks, the outbreak took on pandemic proportions, affecting over 100 countries. It was a significant issue to prevent and control COVID-19 on both national and global scales due to the dramatic increase in confirmed cases worldwide. Government guidelines provide a fundamental resource for communities, as they guide citizens on how to protect themselves against COVID-19, however, they also provide critical guidance for policy makers and healthcare professionals on how to take action to decrease the spread of COVID-19. We aimed to identify the differences and similarities between six different countries’ (US, China, South Korea, UK, Brazil and Haiti) government-provided community and healthcare system guidelines, and to explore the relationship between guideline issue dates and the prevalence/incidence of COVID-19 cases.

**Methods:**

To make these comparisons, this exploratory qualitative study used document analysis of government guidelines issued to the general public and to healthcare professionals. Documents were purposively sampled (*N* = 55) and analyzed using content analysis.

**Results:**

The major differences in the evaluation and testing criteria in the guidelines across the six countries centered around the priority of testing for COVID-19 in the general population, which was strongly dependent on each country’s healthcare capacity. However, the most similar guidelines pertained to the clinical signs and symptoms of COVID-19, and methods to prevent its contraction.

**Conclusion:**

In the initial stages of the outbreak, certain strategies were universally employed to control the deadly virus’s spread, including quarantining the sick, contact tracing, and social distancing. However, each country dealt with differing healthcare capacities, risks, threats, political and socioeconomic challenges, and distinct healthcare systems and infrastructure. Acknowledging these differences highlights the importance of examining the various countries’ response to the COVID-19 pandemic with a nuanced view, as each of these factors shaped the government guidelines distributed to each country’s communities and healthcare systems.

**Supplementary Information:**

The online version contains supplementary material available at 10.1186/s12889-020-09924-7.

## Introduction

The recent outbreak of COVID-19 has led to a major concern of increased mortality in the world. The first outbreak of COVID-19 was reported in Wuhan city, the capital of China’s Hubei Province, in late December, 2019 [1]. Only a few months later, on March 11, 2020, the World Health Organization (WHO) designated the COVID-19 outbreak a pandemic and provided guidelines for COVID-19 case management in the health facility and community. Globally, approximately 3,506,577 confirmed cases of COVID-19 have been reported, including over 247,467 deaths (Johns Hopkins University, May 03, 2020) [2, 3]. The fast spread of the virus reflects how health is connected globally and the necessity of investing in global research efforts to explore, clarify, and address global health emergencies.

When the first COVID-19 outbreak was reported in China, the Chinese government established guidelines that recommended keeping social distance in public places, staying at home, and isolating infected populations to contain the epidemic. After a month, South Korea was assailed by a COVID-19 outbreak. Both governments’ early actions were aggressive in an attempt to stop the virus from spreading, involving both widespread testing for the virus and contact tracing. The COVID-19 response in China and South Korea provided the model for other countries where COVID-19 was just beginning to expand. While it was uncertain whether other countries could implement or adapt the stringent measures endorsed by China and South Korea, the heterogeneous nature of the virus worldwide warranted further investigation into the healthcare and community responses from governments across many nations.

Considering how each country had different capacities, risks, threats, political and socioeconomic challenges, as well as different health care systems, it was unsurprising that each country responded to this threat with different measures and different timings. However, it is also clearly critically important to look at how different countries addressed the first pandemic of coronavirus. Thus, we compared six different countries’ guidelines to investigate their management, incidence, and prevalence of COVID-19 cases. Additionally, we explored the relationship between the guidelines’ issue dates and the prevalence-incidence curves of the different countries.

### Objective

The objective was to compare government guidelines on COVID-19 by six different countries (The United States (US), China, South Korea, The United Kingdom (UK), Brazil and Haiti). This included general public guidelines and healthcare professionals’ (medical institutions) guidelines. We aimed to identify differences and similarities between the countries’ community and healthcare professional guidelines and additionally to explore the relationship between guidelines issue dates and the prevalence/incidence of the COVID-19 cases. This is significant because we can examine how various countries responded to COVID-19 and identify best practices. This approach also allows us to understand how healthcare system and policy capacities shape COVID-19 responses and to share this information to improve responses to COVID-19.

## Methods

### Research design

Our study used document analysis, a standard qualitative research method for evaluating communication and policy research, to explore the differences and similarities between government COVID-19 guidelines from six countries [4, 5]. The following steps were included in the analysis: (i) establishing the document inclusion criteria, (ii) gathering documents, (iii) analyzing key areas, (iv) coding the document, (v) verification, and (vi) analysis [6]. In this approach, the investigators are the primary means of data selection and analysis. This study used purposive sampling to recruit investigators internationally by email and/or phone.

### Country selection

COVID-19 is a global pandemic warranting a cross-national perspective from countries differing on several levels (e.g., geographic region, health and economic resources, stage of COVID-19 spread and response) to maximize range and diversity when exploring the scope of COVID-19-related community and healthcare system guidelines. When selecting the six countries, the following were considered: 1) COVID-19 starting and spiking period, 2) geographic proximity to China, where the COVID-19 spread began, 3) population size, 4) gross domestic product (GDP) status, and 5) eligibility of a bilingual expert in the public health field. For instance, the first COVID-19 outbreak occurred in China in December 2019. Outbreaks in other countries like South Korea, the US, and the UK soon followed in January and February 2020. Finally, in March 2020, Brazil and Haiti noted increased incidence and deaths from the virus. By April 2020, China and South Korea were in the recovery stage of the pandemic while the spread of COVID-19 intensified in countries such as the US, UK, Haiti and Brazil. Geographical differences in the selected countries reflected geographic proximity to China (Korea), largest population in North America (the US), largest population in South America (Brazil), and island countries with significant geographic distance from China (Haiti and the UK). Furthermore, the countries’ population sizes were strongly related to the effectiveness and widespread dissemination of information regarding COVID-19 guidelines [6]. Additionally, the countries’ GDP represented their resources, ability, and strategies employed in response to COVID-19. For instance, at the start of the COVID-19 spread in the UK and Haiti, both countries had very little available resources for COVID testing. With Haiti’s fragile health care system, Haiti did not have the infrastructure to fight the spread of the virus, warranting economic support from other countries. Finally, it was necessary to collaborate with authors who met the following inclusion criteria in order to facilitate document analysis of different countries’ government guidelines: hold a graduate degree, have experience with healthcare material, are fluent in English and have native language fluency (Chinese, Korean, Portuguese, and French/ Haitian Creole) in at least one of the six countries selected.

### Document inclusion/exclusion criteria

Six members of the research team, which consisted of multidisciplinary, cross-cultural researchers, collected the data. The research team only reviewed documents from publicly available government websites for each country (see Additional file 1). Guidelines from government websites were available in different formats, including action and response plans, healthcare and general population guidelines, prevention measures/recommendation flyers, videos, government memos and webpages. Documents or information from nongovernment websites, social media, online newspapers/editorials, peer-reviewed articles, and heath institution guidelines were excluded from the study.

### Data collection

To guide document selection among investigators, a codebook was developed which highlighted the information necessary for each theme (see Additional files 2 and 3). Each document for each country was reviewed to determine the extent to which the document provided answers to at least one of the pre-identified themes (i.e. areas of analysis). The team reviewed government websites weekly for approximately 6 weeks from March 2020–May 2020 to obtain information from government guidelines, and to ascertain whether any new documents were published, or whether old guidelines had been updated. A total of 55 documents (e.g., government guidelines, flyers, memo, webpages) were reviewed to extract data (10 for the US, 3 for China, 10 for South Korea, 8 for the UK, 13 for Brazil, 11 for Haiti) (see Additional file 1). Texts from COVID-19 general public guidelines and government health promotion materials relevant to each pre-identified theme were copied verbatim in their original language and added to an excel spreadsheet. Extracted verbatim texts written in a language other than English (Portuguese, Chinese, Korean, and Haitian Creole/French) were translated to English by bilingual members of the research team and added to the excel spreadsheet to allow for review and analysis of the information as a group (see Additional file 3). Text translation focused primarily on maintaining meaning consistent with health care language rather than cultural nuances, thus, formal translation procedures (e.g., forward and back translation) were not completed. The authors evaluated the English translations, providing constructive feedback about the translation, and confirmed their validity through governmental and professional guidelines and articles.

### Data analysis

Key areas of analysis were articulated in the codebook, which provided a list of codes (e.g., themes and sub-themes) and included six basic components: the code, a brief definition, a full definition, guidelines for when to use or not use the code, and examples of the code. Explicitly, the codebook helped the research team determine the meanings of themes and provided clarity about what to look for within the text of the guidelines. The codes’ sensitivity and specificity were used as a tool for measuring the adequacy of answers to research questions. Data analysis entailed appraising and synthesizing texts from guidelines, which were then organized into major themes and sub-categories through content analysis [7]. Content analysis within the research team was facilitated through online meetings, which were convenient and removed geographic barriers. Researchers assessed the data for coding patterns (e.g., *similarity, differences, frequency)* across different countries regarding their government guidelines for the general public and healthcare professionals. All texts from guidelines were allocated deductively to the a priori themes (deductive codes). During the iterative content analysis process, new themes also emerged (inductive codes). When disagreement ensued during data analysis, the research team recoded, or the primary coder sought advice from another team member for verification and clarification [7]. The original data of the confirmed and deaths cases were downloaded from the Johns Hopkins University Center for Systems Science and Engineering [3]. Tableau, a well-known website for analyzing big data, was used to clean and reshape the data for sharing with the public. The data was then converted to excel files and used to create figures (see Additional file 4) [8].

### Trustworthiness of the data

We established trustworthiness of the data by: 1) focusing on government guidelines, as they are a credible source of information (credibility), 2) using information from guidelines, which maintain dependable and consistent patterns over time and are periodically updated to reflect the evolving understanding of the coronavirus (dependability), 3) using government guidelines, which limited the research team’s bias at the data collection and interpretation level, and improved accuracy with the use of a well-developed codebook *(*confirmability), and 4) analyzing the guidelines of six countries experiencing the coronavirus pandemic at the same time but within different contextual realities (transferability) [9, 10].

## Results

### Theme: Evaluation and testing

#### Sub-them: Screening criteria

When comparing the different government guidelines on screening for signs and symptoms in suspected COVID-19 cases, all countries listed respiratory symptoms as a criterion and the majority – Brazil being the exception – emphasized fever, as well. Interestingly, the US and UK did not list travel history as a criterion. We also noticed that the US and Brazil did not categorize pneumonia as a screening criterion, whereas South Korea, the UK and Haiti emphasized unknown case of pneumonia, clinical or radiological evidence of pneumonia, and bronchopneumonia. A major distinction the authors noted was that only China and the UK specified the detection of suspected COVID-19 cases within the hospital through either radiological evidence via chest X-ray and thoracic Computed Tomography (CT) or lymphocyte counts. Haiti, as of April 20, 2020, expanded the screening criterion from ‘have a fever greater than 38°C within the last 10 days’ to ‘anyone with fever greater than or equal to 38 °C (see Additional file 3).’ Haiti’s screening criteria also included body aches, sudden changes in taste (ageusia) or smell (anosmia), possibility of coming in contact with a healthcare professional diagnosed with COVID-19, or being an occupant of a high risk area while experiencing symptoms compatible with COVID-19 (see Table 1).

**Table 1:** Evaluation & testing; COVID-19 symptoms screening criteria

Countries	Fever	Respiratory symptoms	Pneumonia	Clinical evidence	Contact with confirmed cases	Contact with suspected cases	Travel history	Patients aged over 65 with symptoms	Patients with underlying conditions
**US**	> 100.4 °F / 38 °C	Cough, difficulty breathing	N/A	N/A	N/A	N/A	N/A	With symptoms	With underlying conditions
**China**	Fever	Any respiratory symptoms	Multiple patchy shadows and interstitial changes at the lungs	CT imaging features of COVID-19 / either WBC or lymphocyte count decreases	Within 14 days before the onset of the disease	Within 14 days before the onset of the disease	Within 14 days before the onset of the disease	N/A	N/A
**South Korea**	≥37.5 °C	Coughing, difficulty breathing	Unknow case of pneumonia	N/A	Within 14 days with respiratory symptoms	Within 14 days with respiratory symptoms	Within 14 days with respiratory symptoms	N/A	N/A
**UK (Only for inpatients)**	≥37.8 °C	Persistent cough (with or without sputum), hoarseness, nasal discharge or congestion, shortness of breath, sore throat, wheezing, sneezing	Clinical or radiological evidence of pneumonia	Acute respiratory distress syndrome or influenza	N/A	N/A	N/A	N/A	N/A
**Brazil**	N/A	Coughing, runny nose, difficulty breathing	N/A	N/A	***	Possible patients	Travel abroad in the last 14 days	N/A	N/A
**Haiti**	≥38 °C	Cough with or without respiratory difficulties, headache, body aches, sudden changes in taste and smell	Acute upper respiratory infection with a tendency to develop pneumonia or broncho-pneumonia	N/A	Possibly having had contact with a confirmed COVID-19	Contact with a sick person	Within 14 days with flu symptoms	N/A	N/A

All the information was only extracted from the government’s guidelines. Information from public news or other heath institution guidelines were exclude

The terms were extracted directly from the government guidelines. N/A represents the information did not indicate in the guidelines

*** The Brazil government considers that contact with possible patients would include contact with confirmed cases

Although information about contact with suspected and/or confirmed cases was vital for screening criterion in most countries, the US and UK did not include this. Furthermore, specifically examining symptomatic patients aged over 65 or who had underlying conditions was only found in the US guidelines (see Table 1). Of note, South Korea created a new category in addition to the suspected cases called the Patient Under Investigation (PUI) on April 03, 2020. A PUI is a person who has an epidemiologic link to a collective outbreak of COVID-19 in an area, or a possible contact to a COVID-19 positive person. The US used the term PUI to describe people who exhibit symptoms, or were otherwise suspected of having COVID-19, but had not yet been confirmed via laboratory testing.

### Theme: Evaluation and testing

#### Sub-theme: Screening center types

Across different countries, we identified three different types of screening centers: healthcare facilities, drive-through screening clinics, and walk-through screening clinics. While walk-through screening clinics were available in South Korea, healthcare facilities were the only screening centers available in Brazil and Haiti. Interestingly, Brazil and China did create other satellite facilities for treatment, even though they did not create separate facilities for screening. The US, UK and South Korea conducted drive-through screening clinics.

In particular, South Korea took a distinctly different approach to managing suspected COVID-19 cases. Patients with respiratory symptoms that fit the COVID-19 suspected case criteria were blocked from entering the designated healthcare facilities (called the Public Relief Hospital System) and were redirected to other COVID-19 screening/test centers, or if the hospital was a screening center itself, the patient suspected of having COVID-19 was directed to use a specific entrance for COVID-19 screening before entering the main hospital building. The purpose of establishing the Public Relief Hospital System was to provide safe hospital environments protected against COVID-19 spread. In other words, this was an attempt to block patients with COVID-19 from spreading the virus to general patients who did not have COVID-19.

### Theme: Infection control

#### Sub-theme: General outpatient guidance

Outpatients are patients outside of the hospital who need periodical medical attention due to other morbidities (hypertension, cancer, HIV/AIDS, etc.). In South Korea, outpatients who require healthcare service due to non-COVID-19 diseases were directed to the Public Relief Hospital for follow-up or to see a doctor. These outpatients were strictly separated from patients with any respiratory symptoms.

In Brazil and the US, outpatients were advised to call ahead of their appointment time and were asked whether they had experienced symptoms of respiratory infection. The UK and Haiti avoided treating outpatients in their healthcare facilities. Still, the UK continued with outpatient appointments either through video or phone clinics. Chinese patients, on the other hand, could make an appointment via the phone or online and could then complete their appointment in the hospital as long as the patient made the appointment with a specialist and avoided using the emergency room (ER) or fever clinics where COVID-19 patients had been treated. Haiti did not provide recommendations regarding whether outpatients should make appointments with clinics, and outpatient services were unavailable to the general population. However, Haiti did provide some guidance for those with HIV/AIDS, as Haiti has a high number of individuals suffering from HIV/AIDS. The US updated their guidelines in April 13, 2020, advising healthcare facilities to implement alternatives to face-to-face triage and visits, and instructing patients to utilize cloth face coverings regardless of symptoms upon entry to a healthcare facility. However, the guideline did not specify what alternatives were implemented.

### Theme: Cost support

#### Sub-theme: Cost support

Financial support for testing and treatment was provided mainly or totally by the government in South Korea, the UK, and Brazil. In Haiti, the Haitian government, the US, and the World Bank’s Board of Executive Directors, in conjunction with several international and private organizations, donated money to cover the cost of the country’s COVID-19 response. In China, an individual’s medical cost was subsidized based on the subsidy policy of the local area if the patient was suspected of having COVID-19. However, once the patient received confirmation of COVID-19 infection, the medical cost was subsidized by the authorities. The cost of the clinic visit and testing was made free for all US citizens regardless of insurance status, per The Families First Coronavirus Response Act, which required private and federal insurance to pay for Food and Drug Administration (FDA)-approved testing, and for testing to be free to those who were uninsured. The extent to which the COVID-19 treatment was covered differed between insurance companies.

### Theme: Evaluation and testing

#### Sub-theme: Confirmation of COVID-19

All six countries performed real time PCR to confirm COVID-19 cases. Uniquely, the UK did not provide testing for COVID-19 to the community (at the time of writing), and instead reserved testing for National Health Service (NHS) staff, their relatives and – later in the pandemic – select essential workers. Some unique types of confirmatory lab tests were via virus isolation in South Korea, virus gene sequencing in China, and serological examination in Brazil, Haiti, and the US.

Brazil made the decision to include epidemiological criteria, meaning a confirmed case could be included if the individual met clinical criteria and epidemiological evidence, despite a lack of confirmatory laboratory testing for COVID-19. The US, however, made the distinction that an individual who met those guidelines was considered a probable case. The US also described probable cases as a person meeting the presumptive laboratory evidence and either the clinical criteria or the epidemiological evidence. Finally, an individual could be considered a probable case by the US if their vital records, as in their death certificate, indicated the person died of causes related to COVID-19, despite not having a confirmed laboratory test result.

### Theme: Triage protocols

#### Sub-theme: Hospital admission criteria

All countries’ hospitalization decisions were made on a case-by-case basis. While Haiti’s hospitalization criteria were not specified by the government, Brazil relied on post-collection medical evaluation for hospitalization decisions. The Chinese guideline did not indicate hospital admission criteria. The US recommended hospitalization of people with severe symptoms: septic shock, sepsis, pneumonia, hypoxemic respiratory failure, acute respiratory distress syndrome (ARDS), and cardiomyopathy, etc. The UK required either clinical evidence of pneumonia or radiological evidence with a high suspicion for COVID-19, with ARDS-like or influenza-like symptoms for hospitalization.

Uniquely, South Korea created three different categories, ranging from moderate, severe, to extremely severe for hospitalization. Asymptomatic COVID-19 positive individuals or those with mild symptoms were sent to the Living Treatment Center, a facility that monitored symptoms twice a day and transferred support to the hospital in the event of a worsening of symptom severity.

### Theme: Infection control

#### Sub-theme: Healthcare triage isolation

All six countries developed an isolated area for screening and follow up for symptomatic patients in order to isolate suspected cases. Brazil and the US advised healthcare facilities to place suspected cases in well ventilated spaces that allowed sufficient space between patients. South Korea, Haiti, and China organized their healthcare facilities into different levels of care according to the absence or presence of respiratory symptoms. More specifically, China categorized triage isolation areas into those for confirmed, suspected, or non-COVID-19 patients.

### Theme: Infection control

#### Sub-theme: Visitor access to healthcare facilities

In China, visitors were prohibited from accessing healthcare facilities, whereas the UK and South Korea made exemptions for seriously ill patients receiving end-of-life care, who were allowed one visitor per ward patient. Brazil and Haiti limited the number of visitors to the minimum amount possible, but only Haiti required that all visitors entering the hospital wear a face mask. Although earlier in the pandemic the US made no recommendations regarding visitor access, by April the US Center for Disease Control and Prevention (CDC) advised hospitals to limit the number of visitors allowed.

Except for China’s guidelines, all countries took extra precautions towards visitors, establishing protocols for visitors regarding proper Personal Protective Equipment (PPE) and hygiene. Although the US CDC guidelines were not as restrictive as other countries regarding visitor limitations, the US guidelines suggested actively screening visitors for fever and COVID-19 symptoms upon entry to healthcare facilities. If COVID-19 symptoms were present, the guidelines advised that the visitor not be allowed entry to the facility. Similarly, Brazil suggested avoiding entry of visitors with respiratory symptoms. The US CDC and the Brazilian government also recommended posting visual alerts advising visitors to wash their hands frequently, limiting visitors to the most vulnerable patients (i.e. oncology and transplant awards), encouraging the use of videocall applications in place of in-person visits, and recommending visitors leave the patient during aerosol generating procedures or other specimen collection procedures. Lastly, Brazil and the US instructed visitors to only visit the patient’s room, not any other locations in the facility.

## Community guidelines

### Theme: Prevent getting sick

#### Sub themes: Prevent getting sick

Most recommendations to the community on preventing getting sick were similar between the six different countries. In order to explore the major differences, the sub-themes were organized according to singular actions (i.e. total time washing hands, covering cough and sneezes, face-cover recommendations, etc.).

Generally, face-cover recommendations changed throughout the pandemic, however South Korea and China recommended the use of face masks in public places from the beginning of the pandemic, even if the individual was not sick. The UK did not indicate clear guidance on this matter. The US, Brazil, and Haiti did not initially recommend wearing a face covering, however, the guidelines were updated by the US CDC on April 4th, 2020, by the Brazilian Health Ministry on April 5th, 2020, and by Haiti in the middle of April 2020 to indicate that all people, regardless of whether they were sick, should wear a cloth face covering in public. However, medical grade face masks were still not recommended for the community, as they were to be reserved for health care workers due to shortages.

South Korea, Brazil, Haiti, the US and the UK did not provide guidance on the sharing of personal items in the general community guidelines regarding the prevention of getting sick. China was the only country who mentioned not sharing any personal items to the community as a method for preventing contraction of COVID-19.

Even though most community guidelines on preventing illness recommended maintaining 1.8–2.0 m of physical distance between people to avoid viral transmission, Haiti’s guidance on physical distancing initially recommended staying two steps away from other individuals. Haiti updated their recommendation to staying three steps away from others on April 20th.

As of April 8th, 2020, as a unique measure to prevent viral spread, the South Korean government made it mandatory for all Koreans and long-term stay foreigners who entered South Korea to (1) be tested for COVID-19, (2) install an application on their cell phones: the Self-quarantine Safety Protection App, and (3) abide by the guidelines for self-quarantined persons, including conducting self-diagnosis for a period of 14 days (see Table 2).

**Table 2:** Prevent getting sick

Countries	Total time of washing hands	Hand washing	Cover coughs and sneezes	Social distance	Face cover/gloves	Avoid touching face with unwashed hands
**US**	20 s (until dry if using hand sanitizer	Soap, hand sanitizer (> 60% alcohol)	Elbow or tissue, Immediately wash your hands or use hand sanitizer afterward	6-ft	Cloth face cover (Update on April 4, 2020)	Avoid touching your eyes, nose, and mouth with unwashed hands
**China**	Keep good hand hygiene	Soap, alcohol-based hand sanitizer	Elbow	N/A	Disposable medical facemask, Surgical mask, Gloves are recommended	Avoid touch face with hands when you uncertain about hands’ cleanness.
**South Korea**	More than 30 s	Soap	Elbow	2-m	Facemask	Do not touch your eyes, nose, or mouth with unwashed hands
**UK**	20 s	Soap, hand sanitizer	Tissue, wash your hands	2-m (6-ft)	N/A	Do not touch your eyes, nose, or mouth
**Brazil**	40–60 s with soap, 20–30 s with alcohol	Soap, 70% alcoholic preparation	Tissue or arm	2-m	Face cover (Update on April 5, 2020)	Avoid touching your eyes, nose, and mouth with your unwashed hands
**Haiti**	N/A	Soap	Elbow or disposable handkerchief	2 steps, (Updated on April 20, 2020) 3 steps	Facemask	Remember to always wash your hands before touching your mouth, eyes and nose

All the information was only extracted from the government’s guidelines. Information from public news or other heath institution guidelines were exclude. The terms were extracted directly from the government guidelines. N/A represents the information did not indicate in the guidelines.

### Theme: If you are sick

#### Sub theme: What to do if you are sick

Based on the guidelines, we were able to extract 8 important terms, including *avoid using public transport and crowded places, isolation days and next steps, face mask or cloth face covering, use a separate room or bathroom, sharing household items, sick room ventilation, cleaning instructions, call center for COVID-19* (see Table 3)*.* These terms were ascertained from at least two countries’ guidelines.

**Table 3:** What to do if you are sick

Countries	US	China	South Korea	UK	Brazil	Haiti
**Avoid using public transport and crowded places**	Stay home except to get medical care. Do not visit public areas. Avoid public transportation, ride sharing, or taxis.	Immediately go to the designated medical care institution for having specimen collection and lab analysis and follow the quarantine protocols as requested. Avoid using public transportations and do not go to crowded places	Do not go to school or work avoid outdoor activities	Stay at home and do not meet up with other people, Only go outside for food, health reasons or work (but only if you cannot work from home)	Avoid physical contact with other people, especially the elderly and chronically ill and stay home until you get better	Symptoms of acute respiratory infection should be placed under observation or quarantine residential or institutional quarantine
**Isolation days and next steps**	If have had no fever for at least 72 h without use of fever reducing medication, other symptoms improved, and at least 7 days have passed since symptoms first appeared. OR if no longer have a fever, other symptoms have improved, and receive 2 negative tests in a row, 24 h apart	All family members and close contacts required to take 14-day quarantine	Take a rest at home and monitor the symptoms for 3-4 days Consult with KCDC Call center at 1339, a local code+ 120 or a local health center (visit a triage health center when fever (38 °C) continues, or other symptoms get worse	Stay home 7 days if you have Coronavirus symptoms. After 7 days, if you feel better, you can start your usual routine again.	All residents are in isolation for 14 days	Symptoms of acute respiratory infection should be placed under observation or quarantine residential or institutional quarantine.
**Face mask or cloth face covering**	[Sick person] should wear a cloth face covering, over your nose and mouth if you must be around other people even at home. The caregiver should wear [for cleaning the sick person’s bathroom] a mask/cloth face covering and wait as long as possible after the sick person has used the bathroom	All family members should wear a disposable medical face mask	If necessary [to contact family or others] wear a mask	N/A	The infected person: Wear a mask at all times	Recommended for everyone leaving their home (Update on April 06, 2020)
**Use a separate room or bathroom**	You should stay in a specific “sick room” if possible. Use a separate bathroom if available	N/A	Separate self from others as much as possible. Use a separate bathroom if available (If it is necessary to use a common bathroom, disinfect after use)	N/A	A room Used for isolation. In houses with only one room, other residents must sleep in the living room, away from the infected patient In the room used for insolation, keep the windows open for air circulation. The door must be closed for the duration of the insolation.	N/A
**Sharing household items**	Avoid sharing personal household items: Do not share dishes, drinking glasses, cups, eating utensils, towels, or bedding with other people in your home	N/A	Avoid sharing personal household items (dishes, drinking glasses, utensils, towels, bedding) and wash Used items thoroughly after use	N/A	The waste produced by the contaminated patient needs to be separated and disposed. Bath towels, forks, knives, spoons, glasses and other objects used by the patient. Sofas and chairs cannot be shared either.	N/A
**Sick room ventilation**	N/A	N/A	The isolation room must close the door and open window for ventilation	N/A	In the room used for insolation, keep the windows open for air circulation. The door must be closed for the duration of the isolation.	N/A
**Cleaning instructions**	Wash [household] items thoroughly after use (with soap and water or put in the dishwasher). Clean high-touch surfaces in your isolation area (“sick room” and bathroom) every day; let caregiver clean and disinfect high-touch surfaces in other areas of the home. If a caregiver needs to clean and disinfect a sick person’s bedroom or bathroom, they should do so on an as-needed basis. The caregiver should wear a mask/cloth face covering and wait as long as possible after the sick person has Used the bathroom. Clean and disinfect areas that may have blood, stool, or body fluids on them	[Confirmed COVID-19 person’s] residence, supplies, cloth, beddings, tableware and other belongings have to take the procedure of final disinfection, for future reuse.	If it is necessary to use a common bathroom, disinfect after use. Wash used [household] items thoroughly after use.	N/A	Clean the handle [of the door to the room used for isolation] frequently with 70% alcohol or bleach. Household furniture needs to be cleaned frequently with bleach or 70% alcohol. After using the bathroom, never [skip] washing your hands with soap and water and always clean the toilet, sink and other surfaces with alcohol or bleach to disinfect the environment.	N/A
**Call center for COVID-19**	N/A	N/A	KCDC Call center at 1339, a local code+ 120	N/A	TeleSus 136	Call the Ministry of Public Health’ s center of epidemiology at 4343 3333

All the information was only extracted from the government’s guidelines. Information from public news or other heath institution guidelines were exclude. The terms were extracted directly from the government guidelines. N/A represents the information did not indicate in the guidelines.

Five of the countries recommended people with respiratory symptoms stay at home for certain periods, whereas the Chinese guidelines advised sick people to immediately go to a designated medical care institution for testing, and to then follow the quarantine protocols requested. Each country designated different isolation periods and procedures. As reported by the US and the UK governments, people with respiratory symptoms were to isolate at home and only stop home isolation under the following conditions; no fever for at least 72 h without the use of medications that reduced fever, improvement of other symptoms, and the passage of at least 7 days since symptom onset. Brazil and China advised that, in addition to the person with respiratory symptoms, all family members or fellow residents were to be quarantined for 14 days. In South Korea, any person who had COVID-19 symptoms was mandated to stay at home for at least 3 to 4 days and was then called and given advice by the Korea Centers for Disease Control and Prevention (KCDC) call center.

To prevent the spread of the virus between family/household members, the US, South Korea, and Brazil recommended the ill person be confined to a separate room and bathroom and avoid sharing personal household items. Haiti, China, and the UK did not provide guidance on providing a separate room/bathroom or on sharing personal items.

Isolation room ventilation, such as keeping the window open for air circulation or closing the door, were mentioned in the South Korean and Brazilian guidelines. Cleaning instructions for containing the virus were indicated in different ways in each country, except for in the UK and Haiti. Call centers for COVID-19 were conducted in South Korea, Haiti, and Brazil in the very early stages of the pandemic.

### Theme: If you are sick

#### Sub theme: Threshold to contact a healthcare provider

Across the countries examined, the threshold symptoms for when to contact healthcare providers varied. South Korea advised sick people to contact a healthcare provider if the person had a fever (37.5 °C) or if symptoms worsened. Brazil recommended seeking help if the ill person experienced shortness of breath. The US advised individuals to get medical attention if they experienced persistent pain, chest pressure, cyanosis on lips or face, new confusion, or if unable to be awakened. Haiti mentioned contacting a healthcare provider if the individual had respiratory symptoms. Besides the usual respiratory acute signs (fever, shortness of breath), China also mentioned acute digestive tract symptoms as a reason to reach out to a healthcare facility. In the UK, if a person’s symptoms worsened to the point where they were having difficulty breathing, they were advised to go to the hospital by ambulance facilitated by the online NHS service.

### Theme: If you are sick

#### Sub-theme: Transport to healthcare facilities

The US, South Korea, and China recommended using personal vehicles and to avoid using public transportation to reach healthcare facilities, however South Korea and China specified that individuals should cover their face with a face mask before reaching healthcare facilities. Five of the countries allowed ambulance transport, with the exclusion of China.

## Discussion

WHO acknowledged and announced the impact of COVID-19 on both public health and economic sectors via two interim guidelines published in late March 2020. WHO also emphasized the importance of preparedness for the COVID-19 pandemic, accounting for the countries’ health care capacities [11, 12]. The six countries tend to align with the WHO interim guideline, however there are some differences in the response to the pandemic in each country.

The major differences in evaluation and testing criteria in the guidelines across the six countries centered around the priority of testing for COVID-19 in the population, which strongly depended upon each country’s healthcare capacity including accessibility to healthcare providers, having enough testing kits and reagents, availability of hospital beds, and so on. The most similar recommendations in the evaluation and testing criteria from each government were those pertaining to the clinical signs and symptoms, such as fever and respiratory symptoms, as the priority criteria to initiate COVID-19 testing.

During the writing of this paper, there were no known vaccine or antiviral therapies for COVID-19. Therefore, early detection and diagnostic testing for SARS-CoV-2 were vital to reducing transmission, managing active cases, contact tracing, and understanding epidemiology [13]. The government guidelines concerning screening criteria and capacity for screening – including screening centers, and laboratory testing for COVID-19 in suspected or confirmed cases –were crucial factors in protecting the public from the virus. The WHO criticized countries that had not prioritized testing for COVID-19. Tedros Ghebreyesus, the chief executive of WHO, emphasized the importance of testing by stating, “The most effective way to prevent infections and save lives is breaking the chains of transmission. You cannot fight a fire blindfolded, and we cannot stop this pandemic if we don’t know who is infected. We have a simple message for all countries: test, test, test, test” [14]. However, lack of reagents and/or testing capacity for the SARS-CoV-2 virus challenged all nations included in the study, at least at the beginning of the pandemic. The US, UK, Haiti, and Brazil, in particular, experienced problems with shortages of testing kits for SARS-COV2 due to rapidly increasing demand compounded by national supply chains under stress and national laboratories with limited experience in COVID-19 virus testing [15, 16]. This had a negative impact by potentially obstructing the expansion of COVID-19 testing criteria, resulting in a narrowed range of people undergoing COVID-19 testing, which may have led to increases in the actual number of cases and overall risk of death by COVID-19, but falsely decreased the number of confirmed cases and deaths reported in the nations’ statistics.

**Fig. 1:**
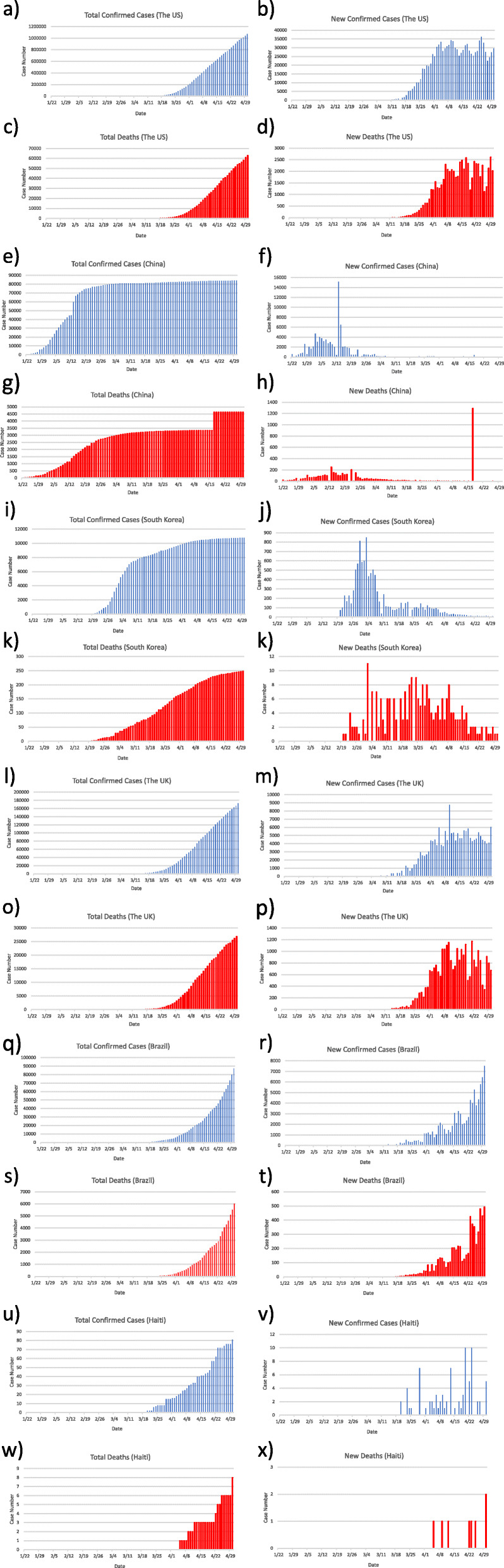
COVID-19 Cases in six countries

According to the UK’s NHS, testing priority was given to 1) intensive care unit patients with suspected coronavirus, 2) patients with severe respiratory illness including pneumonia, 3) isolated cluster outbreaks, and 4) random testing for surveillance purposes [17, 18]. The first 2 confirmed cases occurred in the UK on January 31, 2020 and the first COVID-19 victims died on March 7, 2020 (see Fig. 1). After 20 days, although the UK only tested people who were admitted to hospitals, the number of confirmed cases and disease-related deaths dramatically increased (confirmed cases: 14,745, deaths: 1163) [19]. By April 7, 2020, 1 month after the first COVID-19 deaths, more than 1000 people were dying every day due to viral infection (see Fig. 1). In April 9, 2020, despite the thousands of citizens dying daily due to COVID-19 related causes, the UK government launched large COVID-19 testing centers which prioritized processing samples from health-care workers in self-isolation, allowing them to go back to work [18]. Therefore, people who were not considered a priority, such as non-health care providers or community members with mild respiratory symptoms, were not given access to testing. The limited scope of the UK’s testing approach for COVID-19 was due to a capacity problem, resultant to the consolidation in the number of pathology laboratories nationwide [18]. Many laboratories were centralized, which led to the possibility that each hospital would not necessarily be equipped with a fully functioning lab. This systemic capacity problem may have increased the risk of spread by free movement of people who were suspected of having the disease, since testing was unavailable to those individuals to enforce a stay-at-home order. In the UK, 90,000 people were tested as of the 24th of March - around 1300 COVID-19 tests per million people. Although it was a higher portion than some nations, including the US (around 74 per million as of the 16th of March), it was far behind South Korea (5200 per million as of the 17th of March) [20, 21].

Initially, the US’s CDC included fewer testing criteria than the WHO guidelines. The CDC guidelines recommended testing individuals with a body temperature above 38 °C (fever) and lower respiratory symptoms, those who had a fever and a travel history to China, or those who had a fever and were possibly exposed to a suspected or confirmed COVID-19 case. However, once a patient who did not have any travel history or exposure to any confirmed COVID-19 cases was reported COVID-19 positive, the CDC expanded their testing criterion to include any individuals admitted to a hospital due to lower respiratory symptoms and fever. This addition broadened the spectrum of patients being tested, but also led to rapid increase in the demand for testing.

In February 2020, the CDC acquired, developed, and distributed COVID-19 testing kits to laboratories nationwide, almost one hundred of which reported experiencing several issues with the testing kits. These issues included the failure of negative controls and presentation of inconclusive results. After an internal investigation on February 12, 2020, the CDC reported a faulty reagent as the issue. The CDC immediately recalled all unreliable testing kits and promised to re-manufacture the faulty component and distribute the newly developed reagent to the public health labs as soon as possible. Ultimately, the shortage of COVID-19 test kits at this critical time point possibly interfered with the prevention of increasing confirmed cases early in the outbreak. Furthermore, although the number of confirmed cases and death rate significantly increased each day after March, 20, 2020 in the US (confirmed cases per day around 15,000, deaths per day around 1000), only 97 public health laboratories finished verification and were offering testing on May 6, 2020 [22]. As further evidence of inadequate testing capability, the CDC announced that “although supplies of tests are increasing, it may still be difficult to find a place to get tested” [22].

Together, the capacity for widespread testing and presence of prepared health facilities were key to controlling the dissemination of coronavirus, as evidenced by South Korea. The first COVID-19 incident in South Korea was announced on January 31, 2020, with 7 confirmed cases. The daily confirmed cases remained low for the following month (confirmed cases: 100, deaths: 1) until a super spreader event was initiated on February 29, 2020. Each day for 9 days afterward, the country’s epidemic curve resembled a steep staircase as infections climbed, resulting in dramatically increased confirmed cases and deaths (see Fig. 1). However, by implementing large-scale governmental COVID-19 testing, health officials were able to effectively contact trace and send potentially infected people into quarantine as a preventative measure. By March 25, 2020, more than 357,000 Koreans had been tested. The country reported 10,804 total coronavirus cases and 254 deaths as of May 1, 2020. This was the lowest death rate among the countries examined [3, 23]. Having previously dealt with the Middle East respiratory syndrome (MERS) in 2015, South Korea had already prepared for potential outbreaks of large-scale epidemics, for example by installing negative pressure rooms in hospitals in 2018. Additionally, the country rapidly developed large-scale availability of COVID-19 testing locations, such as K-Walk-Thru and Drive-thru testing stations. These were the first testing centers of their kind in the world and facilitated the quick and safe collection of samples for COVID-19 testing. These unique centers helped not only reduce the risk of cross infections at the in-hospital testing centers, but also increased daily testing capacity amid rapidly rising rates of new cases [24].

WHO emphasized the prioritization of isolated care for patients with higher risk of infection, such as severe and critical illness patients aged over 60 years, and those with underlying medical conditions [25]. Still, exponential escalation in the number of daily confirmed cases placed enormous strain on national medical systems, resulting in limited or total lack of beds for COVID-19 treatment. Therefore, the US, UK, South Korea, Brazil, and Haiti decided patients with mild to moderate coronavirus symptoms should be observed in “Home Isolation”. This approach was a crucial option that only required modification in individual behavior without supplementary expenditure.

Interestingly, China opposed observing mild to moderate coronavirus cases at home, instead directing all potentially infected persons to designated medical care institutions. This policy was initiated in Wuhan, the city where COVID-19 emerged in early February 2020 [26]. On March 27, 2020, more than 60% of coronavirus cases in the country were at Wuhan (see Fig. 1). The city converted exhibition centers and stadiums into shelter hospitals within mere weeks. Epidemiological evidence at the beginning of the pandemic revealed high intrafamily transmission, with 75–80% of all clustered infections diagnosed within families [26, 27]. Quickly emerging alternative hospitals, such as the Fangcang Shelter Hospitals, dedicated to testing and admitting only COVID-19 patients may have led to a reduction in the spread of the virus in the community, thereby decreasing the number of new cases during the pandemic.

On January 22, 2020, the WHO announced the presence of travel-related cases linked to Wuhan City, human-to-human transmission, and reported COVID-19 had been observed outside of China. The WHO strongly advised individuals to report their travel history to their health care providers [28]. However, the UK did not track travel history as it was not considered valuable information in their testing criteria. This was problematic since people who traveled to COVID-19-occurring areas could have potentially acted as carriers of the virus to their respective communities and families, which might have strongly influenced the increasingly steep confirmed case curves. Neither the American, Brazilian nor Haitian governments considered a history of travel to a region of high COVID-19 incidence to be a high priority for testing, or to be an important criterion for suspected cases. Those with a travel history to high spread areas were only encouraged to seek testing if they developed a fever or respiratory symptoms. In direct contrast, the Chinese guidelines suggested that any travelers who traveled to a region or country with occurrence of COVID-19 must be tested, regardless of whether they had developed symptoms.

Although WHO provided a definition of symptoms observed in suspected cases that warranted further surveillance [11], it was a challenge to define the full clinical characteristics of COVID-19. Fever (> 38 °C), breathing problems, and chest radiographs showing bilateral lung infiltrates were the main clinical signs and symptoms reported during the outbreak [13, 29]. For this reason, most countries considered fever, respiratory symptoms, and pneumonia as clinical justification for initiating diagnostic testing. Although by March/April 2020, the UK and US countries were defined as ‘*countries experiencing larger outbreaks*’ (as referred to in Group 4 of the WHO guidelines), they did appear to be largely acting in accordance with WHO advice at that point in time, despite not acting on the previous advice regarding the screening of travelers [11].

Although there was ample evidence of human-to-human transmission, the US and UK did not include contact with confirmed or suspected cases as screening criteria very early in the pandemic. The absence of this criteria early in the pandemic may have led to increased risk of viral spread. In contrast, South Korea undertook an intense contact-tracing program: upon confirmation of a COVID-19 case through laboratory testing, the South Korean government conducted interviews with the infected person, traced their travel history, used GPS phone tracking, and checked their credit-card history. The anonymized data detailing the travel history before diagnosis was published on a public website by the South Korean government. This allowed government officials to quickly release information about potential COVID-19 exposed locations and help people who may have been near those locations make quick decisions on whether they needed to be tested. Though effective, there were and continue to be concerns regarding individual privacy.

With the global spike of COVID-19 and consequent surge in suspected cases and geographic areas affected, the need for implementing screening criteria to better cope with each country’s capacity for screening and laboratory testing became increasingly evident. However, beyond supply chain issues with provision of testing kits, there were significant limitations of the government guidelines for COVID-19 testing in several domains. National health systems and coverage of COVID-19 medical expenses were vital to fostering a sense of financial certainty and a safe environment for those who were infected. Testing and treatment support came mainly or totally from the government in South Korea, the UK, China, and Brazil. All US citizens were covered for FDA-approved COVID-19 testing, regardless of private or federal insurance status, however, treatment coverage was subject to the insurer’s policy. Despite the larger role the governments took in most of the countries examined, Haiti’s COVID-19 health care response was primarily financially supported by the private sector (60%). Hospitals and newly established screening clinics from the private sector worked together with the Haitian Ministry of Health to screen Haitians, however health care facilities from the private sector were not regulated by government officials (hence the paucity of government screening guidelines) [30]. Given these limitations in testing capacity, WHO launched the ‘*COVID-Solidarity Response Fund for WHO’* to support COVID-19 rapid tests for low and middle-income countries [31].

In March 19, 2020, WHO recommended that “when symptomatic, patients are required to wait, ensure they have a separate waiting area” [32]. As an example of the increased preparedness the WHO called for, the South Korean government created temporary ‘*Public Relief Hospitals*’ which provided isolated treatment rooms for patients with respiratory and non-respiratory symptoms to ensure safe medical services to general patients and to prevent viral spread. *Public Relief Hospitals* were divided into two types: Type A and Type B. Both had separate outpatient treatment zones for patients without respiratory symptoms and for patients with respiratory symptoms but differed in whether their testing centers were contained within the hospital. The Korean government also permitted patients who have a chronic disease, but did not have any respiratory symptoms, to receive counseling and prescriptions by telephone or by proxy, therefore decreasing the risk of internal cross-infection within health care facilities for higher-risk patients. This approach was also utilized in the US and UK. In South Korea, non-respiratory patients, such as cancer patients or patients with heart problems, were directed to the general outpatient area at a *Public Relief Hospital*. Patients with mild respiratory symptoms were directed to see a doctor nearby, or to go to a respiratory outpatient area at a *Public Relief Hospital*. Suspected patients or PUI who developed COVID-19 symptoms were referred to a COVID-19 testing center after receiving guidance from a competent clinic or the 1339 call center. Using this triage workflow, Korean hospital systems were better able to prevent internal spreading of the COVID-19 virus in the hospitals and potentially reduced a higher infection-related risk of mortality across the population. The South Korean death rate provided evidence to support this hypothesis, showing that although they had a high rate of confirmed cases (10,780), the total number of deaths was only 250.

WHO recommended healthcare facilities limit the number of visits to suspected or confirmed COVID-19 patients by health care providers, family members, and visitors while being treated in health care facilities. WHO also suggested maintaining a record of all staff and visitors who entered suspected or confirmed COVID-19 patients’ rooms [32]. Even though the US’s federal guidance on hospital visitation seemed more liberal than other countries, especially when contrasted with South Korea and the UK, more restrictions were adopted depending on the local circumstances. For example, although limiting visitors was not advised by the US CDC until April, several hospitals in New York city restricted visitor access as early as March. Brazil’s government strongly recommended individuals with flu or respiratory symptoms are not allowed entry to the hospitals. The government also recommend the hospitals reduce visitor numbers, which, while not mandatory, was heavily implied to be. Although limitations of visitors were not mandatory, wearing a face mask was mandatory for all visitors in Haiti.

The figure illustrates the incidence of confirmed cases and deaths in six countries from January to April.

### Community guidelines

#### Theme: Prevent getting sick

Despite being consistently recommended for use by symptomatic individuals and those in health-care settings, discrepancies were observed in the recommendations on wearing face masks in the general public and community settings. The WHO consistently maintained that the benefits of healthy people using masks in the community setting was not supported by the current evidence, and additionally could contribute to uncertainties or create critical risks [29]. This advice to decision makers remained in place up until the time of this paper submission in May 2020.

Several nations, such as the US and Brazil, changed their face cover recommendations as new studies were conducted that supported the use of face masks as an effective means to limit viral spread. Some studies may under-estimate their protective effects, while observational studies exaggerate them [33]. However, with the emerging evidence of asymptomatic or presymptomatic COVID-19 transmission, the authors note that the community guidance regarding utilizing a face mask and not sharing personal items could significantly prevent potential asymptomatic or presymptomatic transmission, which corroborates other publications [16]. Mask shortages were prevalent across countries in their early stage of use. For example, at the beginning of the pandemic, there was a mask shortage in South Korea due to mass panic-induced purchases by citizens. The South Korean government requested manufacturers increase mask production, and then ensured the newly manufactured masks were directly allocated to pharmacies where only a limited number of masks could be provided to individuals. The number of available masks was displayed in government- and private sector-created apps to prevent citizens from lining up outside pharmacies, which could have resulted in violating physical distancing measures. Additionally, the National Health Insurance Service database showed how many masks were sold to individuals per week.

Generally, the guidance provided across the six nations regarding avoiding infection by washing hands or using alcohol-based hand sanitizer frequently, performing respiratory etiquette when coughing or sneezing, and avoiding touching the face corroborated the WHO guidelines [34].

Despite physical distancing being vital to mitigating the spread of the novel coronavirus, political beliefs affected compliance with COVID-19 social distancing guidelines. This was especially evident in the US, where, in general, people who held contrasting political beliefs to the resident state governing body were less responsive to stay-at-home orders. For example, Republicans did not fully respect and react to stay-at-home orders when Democratic counties announced the order. In a similar fashion, Democrats were less likely to respond to stay-at-home orders when a Republican governor issued the decree [35].

On that point, it is worth noting that although the countries examined all referred to the government issued COVID-19 notices as ‘guidelines,’ these notices were not enforceable equally across countries. As an example, in the US, the CDC’s guidance acted as a framework that could be adapted for use by individual hospitals or by local/state governments for legislative purposes. However, in South Korea the guidelines essentially acted as enforceable legislation with serious financial repercussions.

Another important political development to note occurred in Brazil, when the Ministry of Health included a video on their website focused on clarifying “fake news” about the coronavirus. The video requested users confirm whether information presented in various medias was true before sharing that information with others. It also suggested individuals consult with an official number via WhatsApp for information clarification and communication.

An additional concern was raised regarding the use of health-tracking apps. Various countries used voluntary health-tracking apps to manage the COVID-19 pandemic either for informational, health vigilance, or contact tracing purposes. However, a unique aspect of the South Korean response was the mandate for all Koreans and long-term expatriates to install a health tracking app for contact tracing purposes. Privacy concerns were raised by several publications, some of whom referenced the possibility of preserving data protection [36], while others reflected on the legal implications and the need to refine the data into an aggregate, rather than individual-level data, to better deter the misuse of the data [37].

The countries’ guidelines on how to care for people infected with COVID-19 experiencing mild symptoms at home aligned with the WHO guidance [38]. According to the WHO, ensuring the sick person used a separate room and bathroom in the home would be essential to containing the virus, however, only the US, South Korea, and Brazil made this recommendation to their respective communities. Haiti, the UK, and China did not mention this recommendation in their guidelines. Although those suspected of contracting the coronavirus were requested to stay at home in the UK, limited information was provided to guide the home care process, such as how to disinfect the ill person’s room or how to handle sharing household items in the home. In China, all people suspected of having the coronavirus were instructed to seek testing at a testing center and were admitted to ‘Fangcang Shelter Hospitals.’ Therefore, it could be argued it was not necessary to provide information to the community on how to deal with sick people at home. The decision to advise all people suspected of having the coronavirus to go directly to the hospital is at odds with at least one study, which proposed that instead of guiding the COVID-19 patient to seek healthcare facilities, it would be preferable to provide at-home testing and monitoring [39]. However, while staying at home it is critical to carefully monitor worsening symptoms since medical care is not necessarily immediately available.

The symptom thresholds to contact healthcare providers varied between countries, with a wider array of symptoms (beyond the respiratory types) being included by countries that had dealt with the epidemic for longer periods of time. Clearly a great deal of clinical judgement was necessary for monitoring disease progression, since acting in a timely manner to differentiate a more serious case of COVID-19 was crucial to limiting fatality.

Finally, WHO provided information regarding the transportation of patients with confirmed and suspected COVID-19 to referral health care facilities, however, WHO did not give any information regarding transport mode to individuals with suspected COVID-19 [40]. The guidelines on transportation to healthcare facilities varied in emphasis between governments. A publication from China showed key involvement of public transportation in the dissemination of coronavirus. According to the study, the daily frequency of public transportation entrance and exit from Wuhan was significantly related to the number of COVID-19 cases in other cities [41]. When traveling to a hospital due to the presence of potential COVID-19 symptoms, wearing a face mask, using a personal vehicle, avoiding public transports and/or calling an ambulance were recommended by the Korean, US, and Chinese government’s guidelines. The UK and Haiti advised such patients utilize ambulance transport when heading to the hospital. The Brazilian government did not provided advice regarding transport mode.

### Limitations

These findings are related to the guidelines for healthcare facilities and communities, as updated until April 20, 2020, however some guidelines may have been continuously updated beyond this date. In Haiti, because of the low prevalence of COVID-19 (total confirmed case: 100, deaths: 8 as of May 1, 2020), some information was unable to be obtained from the government guidelines, even though it was provided by news outlets or other medias, which were not included here. This study only used government guidelines accessible by the public, which may have limited the scope of the study’s usable information.

## Conclusion

In summary, all six countries updated their guidelines, especially screening criteria, as the incidence of COVID-19 increased to take more aggressive actions against the progression of COVID-19 spread and to help “flatten the curve,” thus easing some of the burden on the respective healthcare systems. In the initial stages of the outbreak, certain strategies were universally employed to control the deadly virus’s spread, including quarantining the sick, contact tracing, and social distancing. However, these measures would have limited value if the people suspected of contracting the disease were not tested. It is difficult, if not impossible, to identify any one factor as the greatest cause for coronavirus dissemination, but by comparing these countries’ approaches it is possible to identify multiple factors that contribute to an overall effective strategy for reducing its spread. Additionally, there are multiple characteristics that influence the prevalence and incidence of COVID-19, including population density, differences in healthcare infrastructure, and primary means of transportation. Future studies should focus in more detail on these factors and their influence on the prevalence and incidence of COVID-19.

## Supplementary Information

**Additional file 1.** The governments information sources.

**Additional file 2.** Codebook.

**Additional file 3.** Themes list.

**Additional file 4.** Confirmed and Deaths Data.

## Abbreviations

COVID-19: Coronavirus disease
CT: Computed Tomography
CDC: Center for Disease Control and Prevention
ER: Emergency room
FDA: Food and Drug Administration
HIV/AIDS: Human Immunodeficiency Virus/Acquired Immunodeficiency Syndrome
ICU: Intensive care unit
KCDC: Korea Centers for Disease Control and Prevention
MERS: Middle East respiratory syndrome
NIH: National Health Service
PUL: Patient Under Investigation
PPE: Personal Protective Equipment
US: United States
UK: United Kingdom
WHO: World Health Organization
