# Supplementary material for: Comparative analysis of COVID-19 guidelines from six countries: a qualitative study on the US, China, South Korea, the UK, Brazil, and Haiti
Source: BMC Public Health. 2020 Dec 3;20:1853. doi: 10.1186/s12889-020-09924-7 (PMC7711256; doi:10.1186/s12889-020-09924-7)
Supplement: Supplementary file 1 — Additional file 1. The governments information sources. [file 12889_2020_9924_MOESM1_ESM.docx]

Additional file 1. The governments information sources

We accessed the documents between March to May 2020

| **Total US Sources: 10** | | | | | | |
| --- | --- | --- | --- | --- | --- | --- |
| **Intended Audience** | **Source** | | **Reference Title** | **Website** | **Type of Source** | |
| Community | US CDC | | How to Protect Yourself & Others | <https://www.cdc.gov/coronavirus/2019-ncov/prevent-getting-sick/prevention.html> | Guidelines (poster included) | |
|  |  |  | What to Do If You Are Sick | <https://www.cdc.gov/coronavirus/2019-ncov/if-you-are-sick/steps-when-sick.html> | Guidelines (poster included) | |
| Healthcare  Providers |  |  | Evaluating and Testing Persons for Coronavirus Disease 2019 (COVID-19) | <https://www.cdc.gov/coronavirus/2019-nCoV/hcp/clinical-criteria.html> | Guidance on how to prioritize testing for COVID-19 | |
|  |  |  | Interim Infection Prevention and Control Recommendations for Patients with Suspected or Confirmed Coronavirus Disease 2019 (COVID-19) in Healthcare Settings | <https://www.cdc.gov/coronavirus/2019-ncov/hcp/infection-control-recommendations.html> | Guidelines for controlling COVID-19 spread with suspected COVID-19 case | |
|  |  |  | COVID-19 Funding | <https://www.cdc.gov/cpr/readiness/funding-covid.htm> | Summary of Coronavirus Preparedness and Response Supplemental Appropriations Act | |
|  |  |  | Cases in the U.S. | <https://www.cdc.gov/coronavirus/2019-ncov/cases-updates/cases-in-us.html> | Graphical representation of Cases in the US | |
|  |  |  | FAQ: COVID-19 Data and Surveillance | <https://www.cdc.gov/coronavirus/2019-ncov/covid-data/faq-surveillance.html> | FAQ Page | |
|  |  |  | Interim Clinical Guidance for Management of Patients with Confirmed Coronavirus Disease (COVID-19) | <https://www.cdc.gov/coronavirus/2019-ncov/hcp/clinical-guidance-management-patients.html> | Guidelines for managing patients with COVID-19 | |
|  | US FDA | | FAQs on Testing for SARS-CoV-2 | <https://www.fda.gov/medical-devices/emergency-situations-medical-devices/faqs-testing-sars-cov-2> | FAQ Page | |
|  | US Congress | | H.R.6074 - Coronavirus Preparedness and Response Supplemental Appropriations Act, 2020 | <https://www.congress.gov/bill/116th-congress/house-bill/6074/text> | Congressional Law | |
| **Total China Sources: 3** | | | | | | |
| **Intended Audience** | **Source** | | **Reference Title** | **Website** | **Type of Source** | |
| Community | Chinese Center for Disease Control and Prevention | | Prevent getting sick | <http://www.chinacdc.cn/jkzt/crb/zl/szkb_11803/> | Guidelines | |
|  |  |  | What to Do If You Are Sick | <http://www.chinacdc.cn/jkzt/crb/zl/szkb_11803/> | Guidelines | |
| Healthcare  Providers |  |  | Evaluating and Testing | <http://www.chinacdc.cn/jkzt/crb/zl/szkb_11803/> | Guidance | |
|  |  |  | Screening system | <http://www.chinacdc.cn/jkzt/crb/zl/szkb_11803/> | Guidelines | |
|  | National Healthcare Security Administration, National Treasury | | Cost support | <http://www.gov.cn/xinwen/2020-01/30/content_5473177.htm> | Guidelines | |
|  | General Office of the National Health Commission (6th edition) | | COVID-19 prevention and control plan | <http://www.nhc.gov.cn/jkj/s3578/202003/d29e176f35ad4b0a80c74c1d347bfbca.shtml> | Guidelines | |
| **Total South Korea Sources: 10** | | | | | | |
| **Intended Audience** | **Source** | | **Reference Title** | **Website** | **Type of Source** | |
| Community | Central Disease Control Headquarter | | COVID-19 Preventive Actions Together Poster, Coronavirus Disease-19 | <http://ncov.mohw.go.kr/infoBoardView.do?brdId=3&brdGubun=32&dataGubun=321&ncvContSeq=1079&contSeq=1079&board_id=&gubun=> | Guidelines (poster included) | |
|  |  |  | Self-Isolation Guidelines for Families | <http://ncov.mohw.go.kr/duBoardList.do?brdId=2&brdGubun=22&seq=5&#=5> | Guidelines (poster included) | |
| Healthcare  Providers |  |  | Nationally designated inpatient treatment hospital, Coronavirus Disease-19  Patient Treatment & Management, Coronavirus Disease-19 | <http://ncov.mohw.go.kr/en/baroView.do?brdId=11&brdGubun=111&dataGubun=&ncvContSeq=&contSeq=&board_id=>)  <https://www.cdc.go.kr/board/board.es?mid=a20507020000&bid=0019&act=view&list_no=366120&tag=&nPage=1>  <http://ncov.mohw.go.kr/shBoardView.do?brdId=2&brdGubun=21&ncvContSeq=748> | Guidelines | |
|  |  |  | Korean Government’s Response System, Coronavirus Disease-19 | <http://ncov.mohw.go.kr/baroView2.do?brdId=4&brdGubun=42> | Guidelines | |
|  |  |  | **-** Designated Public Relief Hospitals, Coronavirus Disease-19, | <https://www.mohw.go.kr/react/popup_200128.html>  <http://ncov.mohw.go.kr/en/infoBoardView.do?brdId=14&brdGubun=141&dataGubun=&ncvContSeq=1196&contSeq=1196&board_id=&gubun=> | Guidelines | |
|  |  |  | Patient Treatment & Management,  Coronavirus Disease-19 | <http://ncov.mohw.go.kr/baroView3.do> | FAQ Page | |
|  |  |  | Response Coronavirus Disease-19, Nationally designated hospitalization for COVID-19 treatment (5th edition) | <https://www.cdc.go.kr/board/board.es?mid=a20507020000&bid=0019&act=view&list_no=366120&tag=&nPage=1> |  | |
| **Total UK Sources: 8** | | | | | | |
| **Intended Audience** | **Source** | **Reference Title** | | **Website** | | **Type of Source** |
| Community | Public Health England  UK. Gov  Public Health England  UK. Gov  UK. Gov  NHS England  NHS England  NHS England | How to Protect Yourself & Others | | <https://assets.publishing.service.gov.uk/government/uploads/system/uploads/attachment_data/file/874281/COVID-19_easy_read.pdf>  <https://www.gov.uk/coronavirus> | | Guidance |
|  |  | What to Do If You Are Sick | | <https://assets.publishing.service.gov.uk/government/uploads/system/uploads/attachment_data/file/874281/COVID-19_easy_read.pdf> | | Guidelines  (Poster) |
| Healthcare  Providers |  | Evaluating and Testing Persons for Coronavirus Disease 2019 (COVID-19) | | <https://www.gov.uk/government/publications/wuhan-novel-coronavirus-initial-investigation-of-possible-cases/investigation-and-initial-clinical-management-of-possible-cases-of-wuhan-novel-coronavirus-wn-cov-infection> | | Guidance  COVID-19: investigation and initial clinical management of possible cases |
|  |  | Evaluation and testing: Screening Centre Types | | <https://www.gov.uk/government/publications/wuhan-novel-coronavirus-initial-investigation-of-possible-cases/investigation-and-initial-clinical-management-of-possible-cases-of-wuhan-novel-coronavirus-wn-cov-infection> | | Guidance  COVID-19: investigation and initial clinical management of possible cases |
|  |  | Outpatient appointment guidance | | <https://www.england.nhs.uk/coronavirus/wp-content/uploads/sites/52/2020/03/C0044-Specialty-Guide-Virtual-Working-and-Coronavirus-27-March-20.pdf> | | Clinical guide for the management of remote consultations and remote working in secondary care during the coronavirus pandemic |
|  |  | Cost support: Cost support (testing and treatment) | | <https://www.nhs.uk/using-the-nhs/nhs-services/visiting-or-moving-to-england/visitors-who-do-not-need-pay-for-nhs-treatment/> | | Visitors who do not need to pay for NHS treatment |
|  |  | Evaluation and testing: Confirmation of COVID-19 | | <https://www.england.nhs.uk/coronavirus/wp-content/uploads/sites/52/2020/03/clinical-management-of-persons-admitted-to-hospita-v1-19-march-2020.pdf> | | Clinical management of persons admitted to hospital with suspected COVID-19 infection |
|  |  | Triage protocols: Hospital admission criteria | | <https://www.england.nhs.uk/coronavirus/wp-content/uploads/sites/52/2020/03/clinical-management-of-persons-admitted-to-hospita-v1-19-march-2020.pdf> | | Clinical management of persons admitted to hospital with suspected COVID-19 infection |
|  | UK. Gov | Infection control: Healthcare triage isolation | | <https://www.gov.uk/government/publications/wuhan-novel-coronavirus-initial-investigation-of-possible-cases/investigation-and-initial-clinical-management-of-possible-cases-of-wuhan-novel-coronavirus-wn-cov-infection> | | Guidance  COVID-19: investigation and initial clinical management of possible cases |
|  | NHS England | Infection control: Visitor Access to Healthcare facilities | | <https://www.england.nhs.uk/coronavirus/wp-content/uploads/sites/52/2020/03/C0030_Visitor-Guidance_8-April-2020.pdf> | | Visitor guidance |
|  | Public Health England | Infection control: Protection of healthcare personnel | | <https://assets.publishing.service.gov.uk/government/uploads/system/uploads/attachment_data/file/881242/COVID-19_Infection_prevention_and_control_guidance_complete.pdf> | | COVID-19: infection prevention and control guidance |
| **Total Brazil Sources: 13** | | | | | | |
| **Intended Audience** | **Source** | | **Reference Title** | **Website** | **Type of Source** | |
| Community | National Health Surveillance Agency  Ministry of health. | | Guidelines for health services: Measures for prevention and control that must be adopted during assistance to suspected cases or confirmed infection by the new coronavirus (SARS-CoV-2). | <https://www.unasus.gov.br/especial/covid19/pdf/23> | Technical Note on measures for prevention and control | |
|  |  |  | COVID-19 clinical management protocol in Specialized Care | <https://www.unasus.gov.br/especial/covid19/pdf/105> | Clinical management Protocol | |
|  |  |  | **Coronavirus COVID-19: what you need to know and do** | <https://www.saude.gov.br/images/pdf/2020/marco/26/Cartaz-Geral-64x46cm.pdf> | Poster | |
|  |  |  | **Do you have questions about the coronavirus? The Ministry of Health answers you!** | <https://www.saude.gov.br/images/pdf/2020/April/07/Cartilha-Coronavirus-Informacoes-.pdf> | Poster | |
|  |  |  | **Coronavirus. Fake news movie -15** | <https://www.saude.gov.br/images/mp4/2020/marco/19/Filme-Fake-News-15-segundos.mp4> | Video  (The video requests users to confirm whether the information is true before further sharing suggesting consulting with an official number for Whatsapp communication. ) | |
| Healthcare  Providers |  |  | Flowchart for early detection of COVID-19 in a 24-hour UPA emergency care unit and hospital unit not defined as a reference. | <https://www.unasus.gov.br/especial/covid19/pdf/83>  <https://www.unasus.gov.br/especial/covid19/pdf/82> | Poster | |
|  |  |  | Fast-track call center for primary care: fast flow | <https://www.unasus.gov.br/especial/covid19/pdf/59>  <https://www.unasus.gov.br/especial/covid19/pdf/30> | Poster | |
|  |  |  | Coronavirus disease (COE-COVID19) | <https://www.saude.gov.br/boletins-epidemiologicos> | Epidemiological bulletin | |
|  |  |  | Coronavirus COVID-19: Know when and how to do home isolation. | <https://www.saude.gov.br/campanhas/46452-coronavirus> | Poster | |
|  |  |  | Guidelines for Diagnosis and Treatment of COVID-19  Flowchart for care and early detection of COVID-19 in emergency care, 24-hour UPA and hospital unit not designated as a reference | <https://www.unasus.gov.br/especial/covid19/pdf/118>  <https://www.unasus.gov.br/especial/covid19/pdf/40> | Guideline for diagnosis and treatment of COVID-19 (published on April 17)  Poster | |
| **Total Haiti Sources: 11** | | | | | | |
| **Intended Audience** | **Source** | | **Reference Title** | **Website** | **Type of Source** | |
| Community | Ministry of Public Health  World Bank  Ministry of Health | | Prevent getting sick:  MSPP’s COVID-19 Preparedness and Response Plan  GHESKIO in Haiti prepares for COVID-19 | 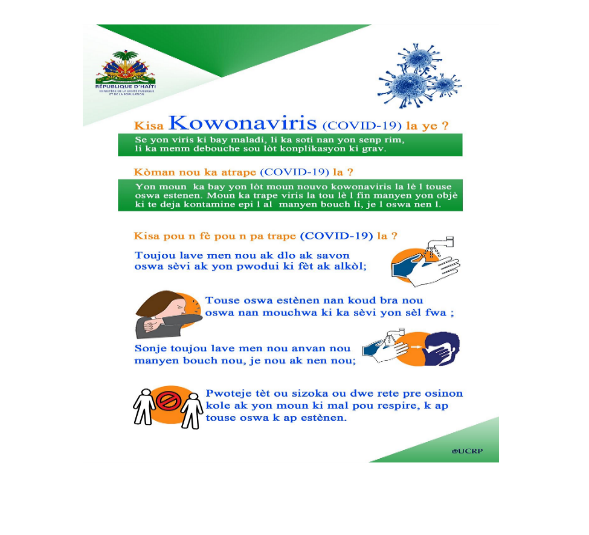 | Poster, this poster had been updated, no longer accessed.  Poster, Guidelines | |
|  |  |  |  | <https://globalhealth.weill.cornell.edu/gheskio-haiti-prepares-covid-19>  <https://www.mspp.gouv.ht/index.php>  <https://mspp.gouv.ht/site/downloads/Nòt%20pou%20laprès.pdf> |  |  |
|  |  |  | What to Do If You Are Sick | <https://mspp.gouv.ht/site/downloads/Nòt%20pou%20laprès.pdf> | PDF, guidelines | |
|  |  |  | COVID-19 Information | <https://ht.usembassy.gov/covid-19-information/> | Guidelines | |
|  |  |  | COVID-19, Haiti in 2020 | <https://mspp.gouv.ht/site/downloads/Sitrep%20COVID-19_09-05-2020%20VC.pdf> | PDF, guidelines | |
| Healthcare  Providers |  |  | MSPP’s COVID-19 Preparedness and Response Plan | <https://www.humanitarianresponse.info/ru/operations/haïti/document/haïti-plan-de-préparation-et-de-réponse-du-mspp-au-coronavirus-mars-2020> | Guidelines | |
|  |  |  | Funding | <https://ht.usembassy.gov/u-s-provides-covid-19-assistance-to-haiti/>  <https://mspp.gouv.ht/site/downloads/2%20nouvo%20ka%20COVID%2019%20konfime%20ak%20yon%20%20moun%20anplis%20ki%20geri%20nan%20peyi%20Dayiti.pdf>  <https://mspp.gouv.ht/site/downloads/57%20Ka%20COVID%2019%20Konfime%20nan%20peyi%20Dayiti.pdf>  <https://www.mspp.gouv.ht/evenement2.php> | Guidelines | |
|  |  |  | Haiti-COVID-19-Strategic-Preparedness-and-Response-Project | <https://www.worldbank.org/en/news/press-release/2020/04/01/world-bank-approves-us20-million-grant-to-support-covid-19-response-in-haiti> | PDF, guidelines | |
