# Supplementary material for: Comparative analysis of COVID-19 guidelines from six countries: a qualitative study on the US, China, South Korea, the UK, Brazil, and Haiti
Source: BMC Public Health. 2020 Dec 3;20:1853. doi: 10.1186/s12889-020-09924-7 (PMC7711256; doi:10.1186/s12889-020-09924-7)
Supplement: Supplementary file 2 — Additional file 2. Codebook. [file 12889_2020_9924_MOESM2_ESM.docx]

**Additional file 2 -Codebook**

| **Guidelines towards** | **Theme** | **Sub-theme** | **Brief Definition** | **Definition** | **When to use** | **When not to use** | **Example:** |
| --- | --- | --- | --- | --- | --- | --- | --- |
| Healthcare providers | Evaluation and testing | Screening criteria | Screening criteria for COVID-19 | Screening signs and symptoms criteria to suspect a COVID-19 case | Apply this code to the list of signs and symptoms that healthcare professionals should consider suspecting a COVID-19 case. | Do not use this code if the signs and symptoms are not listed for tracking COVID-19 cases. | "Symptoms of respiratory infection (for example, cough, runny nose, difficulty breathing); Contact with possible patients with the new coronavirus (SARS-CoV-2); Have travel history to Wuhan and its surrounding areas." |
| Healthcare providers | Evaluation and testing | Screening Center Types | Screening center types | Types of screening places available for COVID-19 | Apply this code to places available for COVID-19 screening. | Do not use this code if the place is not indicated by the guideline to provide screening service. | "Drive-Through Screening Clinics, hospitals, local clinics, primary care facility, healthcare facility" |
| Healthcare providers | Screening system | Outpatient appointment guidance | Outpatient guidance/  management | Guidance and management of individuals who require healthcare service due other non-COVID-19 diseases (outpatient) | Apply this code to guidance provided to healthcare providers and facilities on how to guide and manage individuals who require healthcare service due other non-COVID-19 diseases (outpatient) | Do not use this code if the guidance is for screening or managing patients at the healthcare facility or suspected of COVID-19. | "Pre-check system for respiratory symptoms for all patients, before entering hospitals; These patients should be advised, if possible, to postpone the consultation after the symptoms improve" |
| Healthcare providers | Cost support | Cost support  (Testing and treatment) | Cost support | Available support to cover testing and treatment costs for COVID-19 | Apply this code to descriptions on who will pay the testing and treatment costs for COVID-19. | Do not apply this code to ideas that do not describe who will pay testing and treatment costs for COVID-19. | "The government is providing aid and compensation; pledge to fund the NHS with whatever it needs." |
| Healthcare providers | Evaluation and testing | Confirmation of COVID-19 | Confirmation of COVID-19 | Confirmation and report of COVID-19 cases | Apply this code to guidance related to confirmation and report of COVID-19 cases | Do not apply this code to criteria that does not relate to confirmation and report of COVID-19 cases | "Positive lab test; A COVID19 positive test post swabbing is required to be considered. Clinical symptoms and CxR findings alone are not enough; Real-time PCR" |
| Healthcare providers | Triage protocols | Hospital admission criteria | Hospital admission criteria | Hospital admission criteria for COVID-19 symptomatic individual | Apply this code to guidance related to when symptomatic COVID-19 individuals quarantined at home should be directed to hospital | Do not apply this code to guidance not directed to admit symptomatic COVID-19 patients in the hospital | "Admit patient to the hospital if " |
| Healthcare providers | Infection control | Healthcare triage isolation | Triage isolation | Area for triage isolated in the healthcare facility | Apply this code to guidance related to create a separate area for triage of COVID-19 cases | Do not apply this code to criteria that relate to the organization of a separate area for triage of COVID-19 cases | "Identify a separate, well-ventilated space that allow symptomatic waiting patients to stay away; Type A hospitals have separate outpatient treatment areas for general patients and respiratory patients" |
| Healthcare providers | Infection control | General standard precautions to all healthcare services | General precautions to healthcare services | General standard precaution to all healthcare services | Apply this code to general standard precautions to all healthcare services | Do not apply this code to guidance of precautions not directed to all healthcare services | "All healthcare services should" |
| Healthcare providers | Infection control | Visitor Access to Healthcare facilities | Visitor access | Criteria for allowing healthcare facility visitors to patients and areas where visitors are allowed | Apply this code to guidance on healthcare facility criteria for allowing visitors and which areas of the hospital they are allowed | Do not apply this code to criteria that does not apply for visitors in healthcare facilities | "No access; Visitors must be passively screened for symptoms of acute respiratory illness before entering the healthcare facility" |
| Community | Prevent getting sick | Prevent getting sick | Prevent sickness | Guidelines for the community to prevent getting sick from COVID-19 | Apply this code to guidance for the community on how to prevent getting sick from COVID-19 | Do not apply this code if guidance is not directed to community to prevent getting sick from COVID-19 | "Wash hands 40-60s with soap; Wash hands 20 seconds with soap; cough or sneeze on Tissue or elbow; Do not touch your eyes, nose or mouth if your hands are not clean" |
| Community | If you are sick | If you are sick | Sickness | Guidelines for the community to what to do if the individual is sick | Apply this code to guidance for the community to what to do if the individual is sick | Do not apply this code if guidance is not directed to community what to do if the individual is sick | "Stay at least 14 days at home; Stay in a single bedroom with good ventilation; Wear face mask when leave the room; Sanitize the toilet after use; Avoid using the central air conditioning system. Wear face mask when leave the room. Wash hands before leaving the room; Keep 1-meter distance with other people at home and avoid unnecessary contact. Other family members have to wear face masks;" |
| Community | If you are sick | Threshold to contact healthcare provider | Threshold to contact healthcare provider | Guidelines for the community to what is the threshold symptom to contact healthcare provider | Apply this code to guidance for the community to which signs and symptoms are a threshold to contact the healthcare provider | Do not apply this code if guidance is not directed to guide the community on which signs and symptoms are a threshold to contact the healthcare provider | "If having trouble breathing, or any other emergency warning signs (persistent pain or pressure in the chest, new confusion or inability to arouse, bluish lips or face), or if individual thinks it is an emergency; look for a healthcare service if fever over 37.5C" |
| Community | If you are sick | Transport to healthcare facilities | Transport to healthcare facilities | Guidelines for the community to what transport use when you are sick to reach healthcare facilities | Apply this code to guidance for the community to what transport use when you are sick to reach healthcare facilities | Do not apply this code if guidance is not directed to sick individuals on how to reach healthcare facilities | "personal vehicle with a facemask" |
|  |  |  |  |  |  |  |  |
