# Supplementary material for: Comparative analysis of COVID-19 guidelines from six countries: a qualitative study on the US, China, South Korea, the UK, Brazil, and Haiti
Source: BMC Public Health. 2020 Dec 3;20:1853. doi: 10.1186/s12889-020-09924-7 (PMC7711256; doi:10.1186/s12889-020-09924-7)
Supplement: Supplementary file 3 — Additional file 3. Themes list. [file 12889_2020_9924_MOESM3_ESM.docx]

| Themes / Sub-themes | | | South Korea | | | |
| --- | --- | --- | --- | --- | --- | --- |
|  |  |  | Verbatum | Translation | | |
| Evaluation and testing | Screening criteria | | 1. 발열 (37.5°C 이상) 또는 호흡기증상 (기침, 인후통 등) 이 있으며 최근 14일 이내 중국 (홍콩, 마카오 포함) 을 방문한 자  2.발열 (37.5°C 이상) 또는 호흡기증상 (기침, 인후통 등)이 있으며 최근 14일 이내 확진환자와 밀접하게 접촉한 자  3. 의사의 소견에 따라 신종 코로나바이러스감염증이 의심되는 자  1) 기타 원인불명의 퍠렴 등으로 의사의 판단으로 입원치료가 필요한 자  2) 신종 코로나바이러스감염증 지역사회 유행국가를 여행한 후 14일 이내에 발열 또는 호흡기 증상(기침, 인후통 등)이 나타난 자  <제공: 코로나바이러스감염증-19 사례정의 및 신고 대상, 중앙방역대책본부, 2020.02.8>  Update version on April 02, 2020 is written in English, original text displayed in the translation section. | 1. Fever (37.5°C) or respiratory symptoms within 14 days after visiting China (including Hong Kong and Macau)  2. Patients with confirmed fever (37.5°C) or respiratory symptoms within 14 days of contact with the confirmed patient during the symptom occurrence period  3. Coronavirus infection is suspected, a clinician's judgement  1) Unknown cause of pneumonia, which requires hospitalization according to clinician's judgement  2) Fever (37.5°C) or respiratory symptoms appear within 14 days of visit to the country, region, or country of occurrence.  <Source: Coronavirus Disease-19, Central Disease Control Headquarters, February 8, 2020>  Updated: April 02, 2020  In accordance with the case definitions provided for in these guidelines, patients classified as suspected cases and Patients Under Investigation (PUI) may get tested.  ○ There is no need to get tested out of simple anxiety. We ask that you trust the expert advice of your physicians.  Suspected Cases  A person who develops a fever or respiratory symptoms (coughing, difficulty breathing, etc.) within 14 days of coming into contact with a confirmed patient  Patients Under Investigation  ① A person who is suspected of having the COVID-19 virus as per doctor’s diagnosis of pneumonia of unknown causes.  ② A person who develops a fever (37.5℃ and above) or respiratory symptoms (coughing, difficulty breathing, etc.) within 14 days of travelling overseas  ③ A person with an epidemiologic link to a collective outbreak of COVID-19 in Korea and develops a fever (37.5℃ and above) or respiratory symptoms (coughing, difficulty breathing, etc.) within 14 days.  < Source: Coronavirus Disease-19, Central Disease Control Headquarters, April 02, 2020> | | |
|  | Screening center types | | 1. 국민안심병원 국민들이 코로나19 감염 불안을 덜고, 보다 안심하고 진료 받을 수 있는 안전한 병원체계를 구축  2. 드라이브 스루 검사소  3. 워크스루 검사소 (대규모 진료수, 하루 1000명 검사 가능)  <제공: 코로나바이러스감염증-19, 대한민국 방역체계, 중앙방역대책본부, 2020.02.25> | 1. Established Public Relief Hospital System that can protect and provide a safe hospital environment to patient from COVID-19  2. Dive-through screening clinic  3. Walk-through screening clinic (Large clinic for testing 1000 people per day  < Source: Coronavirus Disease-19, Central Disease Control Headquarters, February 25, 2020> | | |
| Screening system | Outpatient appointment guidance | | Original version is written in English, original text displayed in the translation section.  < Source: Coronavirus Disease-19, Central Disease Control Headquarters, February 24, 2020> | Public Relief Hospitals’ provide segregated treatment for non-respiratory and respiratory patients in order to guarantee medical services to patients in general and prevent the virus from spreading.  Public Relief Hospitals are divided into two types – Type A hospitals have separate outpatient treatment areas for general patients and respiratory patients, while Type B hospitals not only have a separate outpatient area for respiratory patients, but also screening centers capable of collecting samples and dedicated wards for respiratory patients.  Also, the Korean government has permitted non-respiratory patients to receive counseling by phone and prescriptions by phone and by proxy to prevent infection within healthcare institutions.  Non-respiratory patients, such as hypertensive patients or patients with heart problems should go to the general outpatient area at a Public Relief Hospital. Patients with mild respiratory symptoms should go see a physician nearby or go to the respiratory outpatient area at a Public Relief Hospital.  < Source: Coronavirus Disease-19, Central Disease Control Headquarters, February 24, 2020> | | |
| Cost support | Cost support (testing and treatment) | | 한국 정부는 국민들이 감염 예방에 적극 협조하고 생계에 지장을 받지 않도록, 확진환자는 입원 · 치료비, 의심환자 등의 진단검사비는 전액 건강보험 또는 국비로 지원합니다. 또한 자가격리자나 입원 대상자에 대해서는 생활지원비 또는 유급휴가비를 지원하고, 사망시에는 장례비를 지급합니다.<제공: 코로나바이러스감염증-19, 환자치료 및 관리, 중앙방역대책본부> | The Korean government is providing aid and compensation to further encourage the public to actively participate in infection prevention and minimize possible losses inflicted by the virus outbreak. Hospitalization and treatment expenses of confirmed cases and diagnostic testing expenses of suspected cases are entirely covered by National Health Insurance or government expenditure. It is also providing aid for living expenses for those put under self-isolation and awaiting hospitalization and paid leave expenses for their employers, providing aid for funeral expenses for deaths as well.  < Source: Coronavirus Disease-19, Central Disease Control Headquarters> | | |
| Evaluation and testing | Confirmation of COVID-19 | | Original version is written in English, original text displayed in the translation section.  < Source: Coronavirus Disease-19, Central Disease Control Headquarters> | Testing and screening: Novel Coronavirus genetic testing (PCR), virus separation  < Source: Coronavirus Disease-19, Central Disease Control Headquarters> | | |
| Triage protocols | Hospital admission criteria | | Original version is written in English, original text displayed in the translation section.  < Source: Coronavirus Disease-19, Central Disease Control Headquarters> | To hospitalize patients with severe symptoms and provide appropriate treatment options to other confirmed cases, we are classifying patients based on severity and accommodating them at hospitals and living and treatment support centers accordingly  First, confirmed cases are diagnosed at public health centers, and healthcare specialists in city- and province-level patient management teams classify them based on severity (mild, moderate, severe, and extremely severe).  Moderate, severe, and extremely severe cases are immediately hospitalized for treatment (national infectious disease hospitals and other government-designated institutions for hospitalized treatment).  The Living Treatment Center is a quarantine facility mostly for mild or asymptomatic confirmed patients who have been discharged but find it difficult to get treatment at home. The patient will be monitored twice a day and immediately transported to a health care facility if the symptoms get worse. If the symptoms improve, the patient will be checked out in accordance with standards for lifting the quarantine.  Certain state-run facilities and accommodations in each city and province are designated as Living Treatment Centers and are supplied with medical staff, medical equipment (pulse oximetry device, thermometer, blood pressure monitor, CPR kit, chest X-ray radiograph, etc.), individual relief kits (underwear, toiletries, face masks, etc.), and hygiene kits (thermometer and medical supplies).  < Source: Coronavirus Disease-19, Central Disease Control Headquarters> | | |
| Infection control | Healthcare triage isolation | | Original version is written in English, original text displayed in the translation section.  < Source: Coronavirus Disease-19, Central Disease Control Headquarters> | Public Relief Hospitals  Type A hospitals have separate outpatient treatment areas for general patients and respiratory patients, while Type B hospitals not only have a separate outpatient area for respiratory patients, but also screening centers capable of collecting samples and dedicated wards for respiratory patients.  < Source: Coronavirus Disease-19, Central Disease Control Headquarters> | | |
|  | Visitor access to healthcare facilities | | 방문객 통제  -보호자 외의 병문안 등 방문객 전면 통제  -국민안심병원 입원실, 응급실은 보호자 외의 방문객을 전면 통제  환자 보호자는 불가피한 경우에 1명만 -출입이 가능하고, 출입자 명부 작성 등 절차를 거친 뒤에 입원실 또는 응급실 출입  -보호자는 손씻기, 기침 예절 등 감염예방 수칙 준수토록 안내하고 화장실 안내 및 손세정제 비치.<제공: 코로나바이러스감염증-19,중앙방역대책본부> | Visitor control  - All visitation is strictly controlled, and visitors are restricted when entering a hospital.  - The Public Relief Hospital have full control of all visitors to the inpatient ward or emergency care unit. If it is necessary to enter inpatient ward or emergency care unit, only a guardian can enter.  - Guardians should be guided on how to prevent the infection, such as washing their hands. (hand sanitizer should be ready for use)  < Source: Coronavirus Disease-19, Central Disease Control Headquarters> | | |
|  |  | |  | | | |
| Themes / Sub-themes | | | **Brazil** | | | |
|  |  |  | Verbatum | | Translation | |
| Evaluation and testing | Screening criteria | | 1. sintomas de infecção respiratória (por exemplo, tosse, coriza, dificuldade para respirar)  2. ou contato com possíveis pacientes com o novo coronavírus (SARS-CoV-2)." (Source: nota tecnica 04/2020[technical note 04/2020])  3. Viagem nos últimos 14 dias para o exterior? (Source: Fluxograma de hosp de referencia para demanda espontanea) | | 1. Symptoms of respiratory infection (for example, cough, runny nose, difficulty breathing)  2. Contact with possible patients with the new coronavirus (SARS-CoV-2).  3. Travel abroad in the last 14 days? | |
|  | Screening center types | | The Health Ministry issued guidelines towards multiple healthcare facilities of the Brazil's Unified Public Health System (SUS) | | Healthcare facilities from the Brazil's Unified Public Health System (SUS) | |
| Screening system | Outpatient appointment guidance | | Ao agendar consultas ambulatoriais, questione se os pacientes apresentam sintomas de infecção respiratória (por exemplo, tosse, coriza, dificuldade para respirar).  Esses pacientes devem ser orientados, caso seja possível, o adiamento da consulta após a melhora dos sintomas." | | When scheduling outpatient appointments, ask if patients have symptoms of respiratory infection (for example, cough, runny nose, difficulty breathing). These patients should be advised, if possible, to postpone the consultation after the symptoms improve."(Source: nota tecnica 04/2020[technical note 04/2020]) | |
| Cost support | Cost support (testing and treatment) | | Seção II, Art. 196. A saúde é direito de todos e dever do Estado, garantido mediante políticas sociais e econômicas que visem à redução do risco de doença e de outros agravos e ao acesso universal e igualitário às ações e serviços para sua promoção, proteção e recuperação. (Source: CONSTITUIÇÃO DA REPÚBLICA FEDERATIVA DO BRASIL DE 1988)  Art. 3o Os recursos do FNS[Fundo Nacional de Saúde], observado o disposto no art. 2o da Lei no 8.142, de 1990, destinam-se a prover:  I - despesas correntes e de capital do Ministério da Saúde, seus órgãos e suas entidades, da administração direta e indireta, integrantes do SUS;  II - transferências para a cobertura de ações e serviços de saúde destinadas a investimentos na rede de serviços, à cobertura assistencial e hospitalar e às demais ações de saúde do SUS a serem executados de forma descentralizada pelos Estados, pelo Distrito Federal e pelos Municípios;  III - financiamentos destinados à melhoria da capacidade instalada de unidades e serviços de saúde do SUS;  IV - investimentos previstos no plano plurianual do Ministério da Saúde e na Lei Orçamentária Anual;  V - outras despesas autorizadas pela Lei Orçamentária Anual. (Source: DECRETO Nº 3.964, DE 10 DE OUTUBRO DE 2001.) | | Section II, Art. 196. Health is the right of all and the duty of the State, guaranteed through social and economic policies aimed at reducing the risk of illness and other diseases and universal and equal access to actions and services for its promotion, protection and recovery. (Source: CONSTITUTION OF THE FEDERATIVE REPUBLIC OF BRAZIL 1988)  Art. 3 The resources of the FNS [National Health Fund], subject to the provisions of art. 2 of Law 8,142, of 1990, are intended to provide:  I - current and capital expenditures of the Ministry of Health, its organs and entities, of the direct and indirect administration, members of SUS;  II - transfers to cover health actions and services aimed at investments in the service network, healthcare and hospital coverage and other SUS health actions to be carried out in a decentralized manner by the States, the Federal District and the Municipalities;  III - financing aimed at improving the installed capacity of SUS health units and services;  IV - investments provided for in the Ministry of Health's multi-annual plan and in the Annual Budget Law;  V - other expenses authorized by the Annual Budget Law | |
| Evaluation and testing | Confirmation of COVID-19 | | CASOS CONFIRMADOS POR CRITÉRIO LABORATORIAL:caso suspeito de SG ou SRAG com teste de:Biologia molecular (RT-PCR em tempo real, detecção do vírus SARS-CoV2, Influenza ou VSR):oDoença pelo Coronavírus 2019:com resultado detectável para SARS-CoV2.oInfluenza:com resultado detectável para Influenza.oVírus Sincicial Respiratório:com resultado detectável para VSR.Imunológico (teste rápido ou sorologia clássica para detecção de anticorpos):oDoença pelo Coronavírus 2019:com resultado positivo para anticorpos IgM e/ou IgG. Em amostra coletada após o sétimo dia de início dos sintomas.POR CRITÉRIO CLÍNICO-EPIDEMIOLÓGICO:caso suspeito de SG ou SRAG com:Histórico de contato próximo ou domiciliar, nos últimos 7 dias antes do aparecimentodos sintomas, com caso confirmado laboratorialmente para COVID-19 e para o qual não foi possível realizar a investigação laboratorial específica.CASO DESCARTADO DE DOENÇA PELO CORONAVÍRUS 2019 (COVID-2019)Caso suspeito de SG ou SRAG com resultado laboratorial negativo para CORONAVÍRUS (SARS-COV-2 não detectável pelo método de RT-PCR em tempo real), considerando a oportunidade da coletaOUconfirmação laboratorial para outro agente etiológico. (Source: Diretrizes para Diganóstico de Tratamento da COVID-19) | | CASES CONFIRMED BY LABORATORY CRITERIA: suspected case of SG or SRAG with a test of: Molecular biology (RT-PCR in real time, detection of SARS-CoV2, Influenza or VSR): o Coronavirus 2019: with detectable result for SARS- CoV2.oInfluenza: with detectable result for Influenza. Respiratory Syncytial Virus: with detectable result for RSV. • Immunological (rapid test or classic serology for antibody detection): o Coronavirus 2019: with positive result for IgM and / or IgG antibodies. In a sample collected after the seventh day of symptom onset. BY CLINICAL-EPIDEMIOLOGICAL CRITERIA: suspected case of SG or SRAG with: History of close or home contact, in the last 7 days before the onset of symptoms, with a laboratory confirmed case for COVID-19 and for which it was not possible to carry out the specific laboratory investigation. DISCARDED DISEASE BY CORONAVIRUS 2019 (COVID-2019) Suspected case of SG or SRAG with negative laboratory result for CORONAVIRUS (SARS-COV-2 not detectable by the RT- Real-time PCR), considering the opportunity of collecting OR laboratory confirmation for another etiologic agent. (Source: COVID-19 Treatment Diagnostic Guidelines) | |
| Triage protocols | Hospital admission criteria | | Realizar avaliação médica pós coleta (Source:Fluxograma para atendimento e detecção precoce de COVID-19 em hospital de referência para indivíduos por demanda espontânea) | | Perform post-collection medical evaluation | |
| Infection control | Healthcare triage isolation | | Identifique um espaço separado e bem ventilado que permita que os pacientes sintomáticos em espera fiquem afastados e com fácil acesso a suprimentos de higiene respiratória e higiene das mãos.  Estes pacientes devem permanecer nessa área separada até a consulta ou encaminhamento para o hospital (caso seja necessária a remoção do paciente).  (Source: nota tecnica 04/2020[technical note 04/2020]) | | Identify a separate, well-ventilated space that allow symptomatic waiting patients to stay away [from each other] and easily access to respiratory hygiene and hand hygiene supplies. These patients should remain in that separated area until consultation or referral to the hospital (if removal of the patient is necessary). | |
|  | Visitor access to healthcare facilities | | Considerando-se a pandemia da Covid-19 e com o objetivo de garantir segurança no atendimento aos pacientes, a integridade dos acompanhantes, visitantes e trabalhadores do serviço de saúde, assim como a prevenção de infecções, orienta-se: •Em serviços de saúde estabelecidos como referência ou retaguarda para atendimento aos pacientes com Covid-19, suspender as visitas sociais a estes pacientes. Caso o serviço não possua fluxo diferenciado para circulação dos demais pacientes e acompanhantes, recomenda-se a suspensão de todas as visitas. •Caso o serviço de saúde opte por manter uma rotina de visitas, deve-se reduzir a circulação das pessoas, o número de visitantes e estabelecer horários para sua realização, além de designar sala de espera ampla e ventilada separada dos demais atendimentos. •Conversar com a família sobre a possibilidade de se manter um único acompanhante para o paciente durante o período de internação sendo este com idade entre 18 e 59 anos, sem doenças crônicas ou agudas. Ressalta-se que acompanhantes com faixa etária de risco maior para a Covid-19 ou com antecedentes de doenças crônicas/imunossupressão não devem estar na condição de acompanhantes. •Proibir acompanhantes para os pacientes com síndrome gripal (exceto em condições previstas por lei: crianças, idosos e portadores de necessidades especiais). •Evitar a entrada de acompanhantes/visitantes com sintomas respiratórios. •Recomenda-se evitar visitas e acompanhantes a pacientes em unidade de terapia intensiva (UTI); Revezamentos de acompanhantes somente se necessário. •Visitantes ou acompanhantes deverão evitar contato direto com o paciente. Caso seja necessário e haja possibilidade de contato com fluidos corporais, deverão ser fornecidas luvas e orientar higiene das mãos sempre que tocar o paciente.•Solicitar a saída do acompanhante do quarto/enfermaria em caso de procedimentos geradores de aerossol. •Seguir rigorosamente as principais medidas preventivas do Ministério da Saúde: lavar as mãos com água e sabão, na sua ausência, usar álcool em gel a 70%; cobrir nariz e boca com lenço descartável ao tossir ou espirrar – quem não o tiver, deve usar o antebraço como barreira, e não as mãos, para evitar tocar em locais que possam contaminar outras pessoas; evitar aglomerações; manter os ambientes bem ventilados; e não compartilhar objetos pessoais.Importante: Cada serviço de saúde tem autonomia para orientações específicas, considerando suas características próprias e as recomendações supracitadas de acordo com as orientações da Comissão de Controle de Infecção Hospitalar (CCIH). (Source:Protocolo de Manejo Clinico da COVID-19 na Atenção Especializada) | | Considering the Covid-19 pandemic and with the objective of guaranteeing safety in patient care, the integrity of the companions, visitors and health service workers, as well as the prevention of infections, is oriented: • In health services established as a reference or rearguard for care for patients with Covid-19, suspend social visits to these patients. If the service does not have a different flow for circulation of other patients and companions, it is recommended to suspend all visits. • If the health service chooses to maintain a routine of visits, it is necessary to reduce the circulation of people, the number of visitors and establish timetables for their performance, in addition to designating a large and ventilated waiting room separate from other services. • Talk to the family about the possibility of keeping a single companion for the patient during the hospitalization period, aged between 18 and 59 years, without chronic or acute illnesses. It should be noted that companions with a higher risk age group for Covid-19 or with a history of chronic diseases / immunosuppression should not be in the condition of companions. • Prohibit companions for patients with the flu syndrome (except under legal conditions: children, the elderly and people with special needs). • Avoid the entry of companions / visitors with respiratory symptoms. • It is recommended to avoid visits and companions to patients in the intensive care unit (ICU); Accompanying relays only if necessary. • Visitors or companions should avoid direct contact with the patient. If necessary and there is a possibility of contact with body fluids, gloves and hand hygiene should be provided whenever the patient is touched • Request the companion to leave the room / ward in case of aerosol generating procedures. • Strictly follow the main preventive measures of the Ministry of Health: wash your hands with soap and water, in your absence, use 70% gel alcohol; cover nose and mouth with disposable handkerchief when coughing or sneezing - those who do not have it should use their forearms as a barrier, not their hands, to avoid touching places that could contaminate other people; avoid agglomerations; maintain well-ventilated environments; and not sharing personal objects. Important: Each health service has autonomy for specific guidelines, considering its own characteristics and the above recommendations according to the guidelines of the Hospital Infection Control Commission (CCIH).  (Source: COVID-19 Clinical Management Protocol in Specialized Care) | |
|  |  | |  | | | |
| Themes / Sub-themes | | | **China** | | | |
|  |  |  | Verbatum | Translation | | |
| Evaluation and testing | Screening criteria | | 1. 流行病学史  1）发病前14天内有武汉市及周边地区，或其他有病例报告社区的旅行史或居住史  2）发病前14天内与新冠病毒感染者（核酸检测阳性）有接触史  3）发病前14天内曾接触过来自武汉市及周边地区，或来自有病历报告社区的发热或有呼吸道症状的患者  4）聚集性发病。两周内在小范围内，如家庭，办公室，学校班级等场所，出现两例及以上发热和呼吸道症状的病历。  2. 临床表现  1）发热或呼吸道症状  2）具有新型冠状病毒肺炎影像学特征  3）发病早期白细胞总数正常或降低， 淋巴细胞计数减少  *有流行病学史中的任何一条，且符合临床表现中的任意两条。或无明确流行病学史，符合临床表现中的三条。  《国家卫生健康委办公厅》印发《新型冠状病毒肺炎防控方案》第六版 | Epidemiological History  1. Have travel history to Wuhan and its surrounding areas, or to communities which have infected cases reported within 14 days before the onset of the disease  2. Have a history of contact with those infected with Covid-19 (those with a positive NAT result) within 14 days before the onset of the disease;  3. Have a history of contact with patients with respiratory symptoms or fever within 14 days before the onset of the disease  4. Disease clustering (2 or more cases with fever and/or respiratory symptoms occur at such places as homes, offices, school classrooms, etc. within 2 weeks).  Clinical Manifestations  1. Have fever or respiratory symptoms.  2. The patient has the following CT imaging features of COVID-19: multiple patchy shadows and interstitial changes occur early, particularly at the lung periphery. The conditions further develop into multiple ground-glass opacities and infiltrates in both lungs. In severe cases, the patient may have lung consolidation and rare pleural effusion;  3. The white blood cells count in the early stage of the disease is normal or decreased, or the lymphocyte count decreases overtime.  * Coincide with one item of epidemiological section and at least with two items of clinical section; Or match all three items of clinical section and no item of epidemiological section.  Source: <COVID-19 prevention and control plan> given by the General Office of the National Health Commission (6^th^ edition) | | |
|  | Screening center types | | 1. 当地医院，社区诊所，军队医院  2. 当地疾病防控中心  3. 第三方测试机构 | 1. Healthcare Facilities (Public hospitals, Community hospitals, Military hospitals)  2. Local Quarantine Station and Center for Disease Control  3. Third Testing Organization | | |
| Screening system | Outpatient appointment guidance | | (1)原则上尽可能少去或不去医院,除非必须立即就医的急症、危重症患者。如果必须去就医,应就近选择能满足需求的、门诊量较少的医疗机构;如果必须去医院,公众只做必须的、急需的医疗检查和医疗操作,其他项目和操作尽可能择期补做;如果可以选择就诊科室,尽可能避开发热门诊、急诊等诊室。  (2)若需前往医院,尽可能事先网络或电话了解拟就诊医疗机构情况,做好预约和准备,熟悉医院科室布局和步骤流程,尽可能减少就诊时问。  (3)前往医院的路上和在医院内,患者与陪同家属均应该全程佩戴一次性使用医用口罩。  (4)如果可以,应避免乘坐公共交通工具前往医院。  (5)随时保持手卫生,准备便携含酒精成分的免洗手消毒剂。在路上和医院时,人与人之间尽可能保持距离(至少1米)。  (6)若路途中污染了交通工具,建议使用含氯消毒剂和过氧乙酸消毒剂,对所有被呼吸道分泌物或体液污染的表面进行消毒。  (7)尽量避免用手接触口、眼、鼻,打喷嚏或咳嗽时用纸巾或肘部遮住口、鼻。  (8)接触医院门把手、门帘、医生白大衣等医院物品后,尽量使用手部消毒液,如果不能及时手部消毒,不要接触口、眼、鼻。医院就诊过程中,尽可能减少医院停留时间。  (9)患者返家后,立即更换衣服,流水认真洗手,衣物尽快清洗。  (10)若出现可疑症状(包括发热、干咳、乏力、鼻塞、流涕、咽痛、肌痛和腹泻等症状),根据病情及时就诊,并向接诊医师告知过去2周的活动史。  <中国疾病预防控制中心> <http://www.chinacdc.cn/jkzt/crb/zl/szkb_11803/> | 1. Generally, avoid visiting hospital as possible as you can, unless emergency occurs. Choose the nearby medical care center with fewer traffic, if medical service required. Only take the essential and necessary medical services in hospital, supplementary tests and complementary treatments shall be implemented in future. Make appointment with specialty if possible, avoid visiting the ER and fever clinics for help.  2. Make appointment online or make a phone call to get the knowledge of the hospital situation ahead of arrival. Be familiar with the hospital layout and visit procedure for the purpose of diminish the total time of visit.  3. Both the patient and company must wear face mask on the way to the hospital, and during the hospital visit.  4. Avoid taking public transportation to go to hospital, if possible.  5. Carry the alcohol-based hand sanitizer to keep good hand hygiene anytime. Keep at least 1-meter distance with others while on the road and in hospital.  6. If the vehicle gets contaminated, disinfect the surface that has been contaminated by body fluids or discharge, via using either chlorine-containing disinfectant or peracetic acid disinfectants.  7. Do not touch your mouth, eyes and nose by hands. Use elbow or tissue to cover your mouth when you sneeze.  8. Use hand sanitizer after touching the door noob, curtain and doctor's white coat in hospital. If you cannot sanitize hands, do not touch your face. Diminish the total time of staying in hospital.  9. Change cloth, wash hands carefully after the patient get back home. Laundry the contaminated cloth asap.  10. If any suspicious symptoms occur (include fever, dry cough, fatigue, nasal congestion, running nose, sore throat, muscle pain, diarrhea, etc.), visit hospital in time and report your activity history of past 2 weeks to your doctor.  Source: <Chinese Center for Disease Control and Prevention>  <http://www.chinacdc.cn/jkzt/crb/zl/szkb_11803/> | | |
| Cost support | Cost support (testing and treatment) | | 国家医疗保障局、财政部联合印发《关于做好新型冠状病毒感染的肺炎疫情医疗保障的通知》,明确规定对确诊为新型冠状病毒肺炎患者发生的医疗费用,实施综合保障,个人负担部分由财政给予补助。《关于做好新型冠状病毒感染的肺炎疫情医疗保障作的补充通知》要求,疑似患者(含异地就医患者)发生的医疗费用,个人负担部分由就医地制定财政补助政策并安排资金,实施综合保障,中央财政视情况给予适当补助.  <国家财政部>，<国家财政局>联合印发  <http://www.gov.cn/xinwen/2020-01/30/content_5473177.htm> | National Healthcare administration and National Treasury jointly post the <Notice about the Covid-19 epidemic medical care>. It explicitly stipulates that the medical cost for individual payment can be subsidized by authority, once the patient be confirmed infection. <Supplementary Notice about the Covid-19 epidemic medical care> requires that the medical cost for individual payment can be subsidized base on the subsidy policy of local area, for all suspicious patients. And the central government will attribute appropriately.  <National Healthcare Security Administration> & <National Treasury> post jointly  <http://www.gov.cn/xinwen/2020-01/30/content_5473177.htm> | | |
| Evaluation and testing | Confirmation of COVID-19 | | 符合疑似病例标准的基础上， 具备以下病原学证据之一，可以确诊：  1. 实时荧光RT-PCR 检测新型冠状病毒核酸阳性。  2. 病毒基因测序， 与已知的新型冠状病毒高度同源  《国家卫生健康委办公厅》印发《新型冠状病毒肺炎防控方案》第六版 | Confirmation Criteria:  1. The Real-Time fluorescence- PCR shows positive result on Covid-19 nucleic acid  2. Virus gene sequencing. The result is highly homologous with Covid-19 gene sequence.  Source: <COVID-19 prevention and control plan> given by the General Office of the National Health Commission | | |
| Triage protocols | Hospital admission criteria | | Not indicated on the government guidelines.  This information is not indicated on the guideline, however, a published article, which cited all different resources from government information, mentioned ‘Chinese policy makers decided against home isolation of patients with mild to moderate COVID-19’.  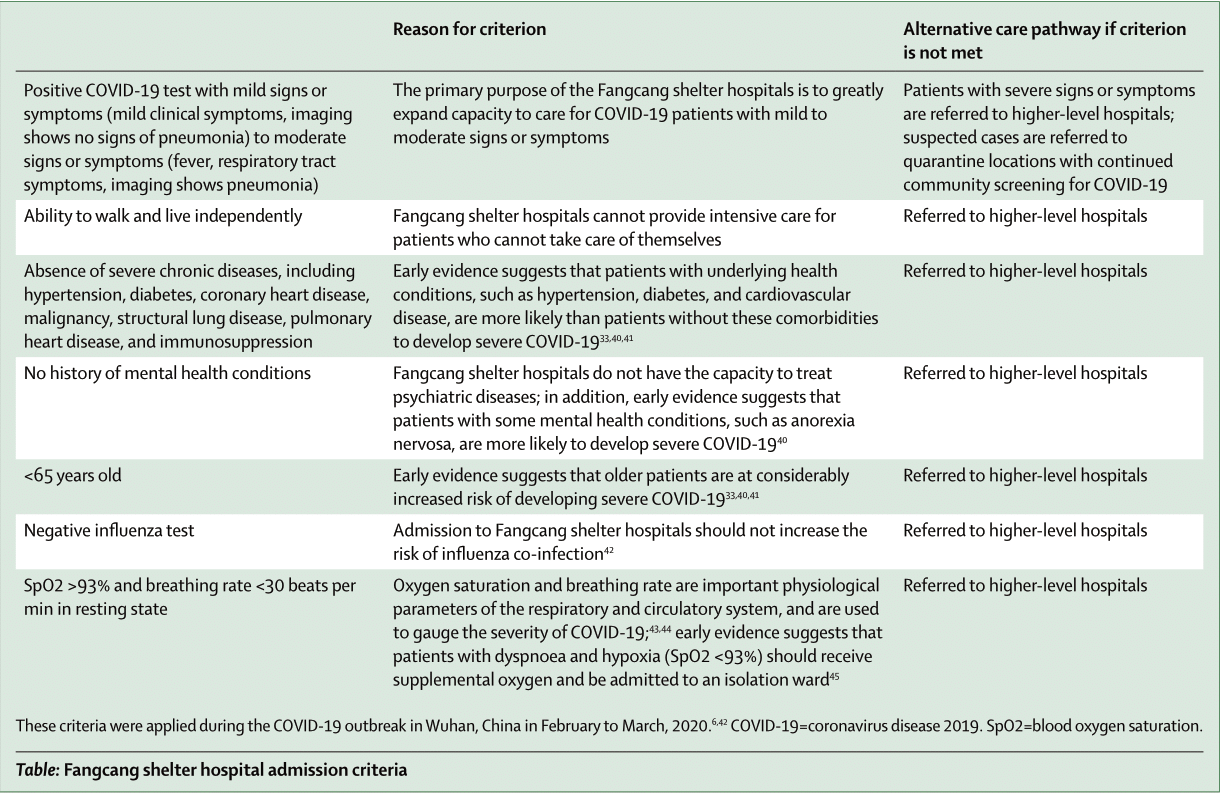  Source: <Chen, S., Zhang, Z., Yang, J., Wang, J., Zhai, X., Bärnighausen, T., & Wang, C. (2020). Fangcang shelter hospitals: a novel concept for responding to public health emergencies. *The Lancet*.> | | | |
| Infection control | Healthcare triage isolation | | 1）医疗机构应设相对独立的发热门诊，医院入口处有发热门诊专用单向通道且有明显标识；  2）人员流向按照“三区两通道”原则，设有污染区、潜在污染区、清洁区，分区明确，污染区与潜在污染区之间设置两个缓冲区；  3）设置独立污物通道；设置可视传递间进行办公区（潜在污染区）向隔离病房（污染区）的单向物品传递；  4）应制定医务人员穿脱防护用品的流程、按区域步骤制作流程图和配置穿衣镜，严格遵守行走路线；  5）配备感染防控技术人员督导医务人员防护用品的穿脱，防止污染；  6）在污染区的所有物品未经消毒处理，不得带离污染区域。  7）设置独立的检查室、化验室、留观室、抢救室、药房、收费处等；  8）设置预检分诊处，对患者做好初步筛查；  9）对诊疗区域进行分区：有流行病学接触史且伴有发热及或呼吸道症状，进入新冠疑似区域；无明确流行病学接触史的进入普通发热患者区域。  10）疑似患者和确诊患者分病区安置；  11）疑似患者单人单间，病室内配备有独立卫生间等生活设施，确保患者活动范围固定于隔离病室内；  12）确诊患者可同病室安置，床间距≥1.2米，病室内配备有独立卫生间等生活设施，确保患者活动范围固定于隔离病室内。  <新冠肺炎防治手册> 浙江大学医学院附属第一医院临床经验 | (1) Healthcare facilities shall set up a relatively independent fever clinic including an exclusive one-way passage at the entrance of the hospital with a visible sign;  (2) The movement of people shall follow the principle of "three zones and two passages": a contaminated zone, a potentially contaminated zone and a clean zone provided and clearly demarcated, and two buffer zones between the contaminated zone and the potentially contaminated zone;  (3) An independent passage shall be equipped for contaminated items; set up a visual region for one-way delivery of items from an office area (potentially contaminated zone) to an isolation ward (contaminated zone);  (4) Appropriate procedures shall be standardized for medical personnel to put on and take off their protective equipment. Make flowcharts of different zones, provide full-length mirrors and observe the walking routes strictly;  (5) Infection prevention and control technicians shall be assigned to supervise the medical personnel on putting on and removing protective equipment so as to prevent contamination;  (6) All items in the contaminated zone that have not been disinfected shall not be removed.  (7) Set up an independent examination room, a laboratory, an observation room, and a resuscitation room;  (8) Set up a pre-examination and triage area to perform preliminary screening of patients;  (9) Separate diagnosis and treatment zones: those patients with an epidemiological history and fever and/or respiratory symptoms shall be guided into a suspected COVID-19 patient zone; those patients with regular fever but no clear epidemiological history shall be guided into a regular fever patient zone.  (10) Suspected and confirmed patients shall be separated in different ward areas;  (11) Suspected patients shall be isolated in separated single rooms. Each room shall be equipped with facilities such as a private bathroom and the patient's activity should be confined to the isolation ward;  (12) Confirmed patients can be arranged in the same room with bed spacing of not less than 1.2 meters (appx 4 feet). The room shall be equipped with facilities such as a bathroom and the patient's activity must be confined to the isolation ward.  Source: Liang, Tingbo. "Handbook of COVID-19 prevention and treatment." *The First Affiliated Hospital, Zhejiang University School of Medicine. Compiled According to Clinical Experience* (2020). | | |
|  | Visitor access to healthcare facilities | | 谢绝家属探视和陪护，患者可携带电子通信设备与外界沟通 | Family visits and care shall be declined. Patients should be allowed to keep  electronic communication devices to facilitate interactions with others; | | |
|  |  | |  | | | |
| Themes / Sub-themes | | | **U.S.A.** | | | |
| Evaluation and testing | Screening criteria | | As of March 24th  Clinicians should use their judgment to determine if a patient has signs and symptoms compatible with COVID-19 and whether the patient should be tested. Most patients with confirmed COVID-19 have developed fever (may be subjective or confirmed) and/or symptoms of acute respiratory illness (e.g., cough, difficulty breathing).  Priorities for testing include (in order from biggest priority to least):  1. Ensuring optimal care for all hospitalized patients with symptoms and systematic healthcare workers who have been exposed to maintain the integrity of the healthcare system  2. Ensure that those who are at highest risk of complications of infection are rapidly ID'd and appropriately triaged (patients 65+ with symptoms, patients with underlying conditions)  3. As resources allow, test individuals with symptoms in communities with rapid spread and increasing hospital cases  4. Individuals without symptoms  Source: https://www.cdc.gov/coronavirus/2019-nCoV/hcp/clinical-criteria.html | | | |
|  | Screening center types | | As of April 5th  1. Healthcare Facilities (public health, commercial, and healthcare system/academic laboratories using an Emergency Use Authorization authorized test)  2. Drive Through Screening Clinics  Source: https://www.fda.gov/medical-devices/emergency-situations-medical-devices/faqs-testing-sars-cov-2 | | | |
| Screening system | Outpatient appointment guidance | | As of March 19th  1. When scheduling appointments for routine medical care (e.g., annual physical, elective surgery), instruct patients to call ahead and discuss the need to reschedule their appointment if they develop symptoms of a respiratory infection (e.g., cough, sore throat, fever )  2. When scheduling appointments for patients requesting evaluation for a respiratory infection or COVID-19, use nurse directed triage protocols to determine if appointment is necessary or if patient can be managed from home.  3. If the patient must come in for an appointment, instruct them to call beforehand to inform triage personnel that they have symptoms of a respiratory infection and to take appropriate preventive actions (e.g., follow triage procedures, wear a facemask upon entry and throughout their visit).  Additional Considerations:  -Cancel group healthcare activities (e.g., group therapy, recreational activities).  -Postpone elective procedures, surgeries, and non-urgent outpatient visits.  Update April 13th:  When scheduling appointments for routine medical care (e.g., annual physical, elective surgery), instruct patients to call ahead and discuss the need to reschedule their appointment if they develop fever or symptoms of COVID-19 on the day they are scheduled to be seen. Advise them that they should put on their own cloth face covering, regardless of symptoms, before entering the facility.  Additional Considerations:  Implement alternatives to face-to-face triage and visits.  Cancel group healthcare activities (e.g., group therapy, recreational activities).  Postpone elective procedures, surgeries, and non-urgent outpatient visits.  Source: https://www.cdc.gov/coronavirus/2019-ncov/hcp/infection-control-recommendations.html | | | |
| Cost support | Cost support (testing and treatment) | | Coronavirus Preparedness and Response Appropriations Act  -Signed into law on March 6, 2020  -Provides $8.3 billion in total funding  -$2.2 billion to the CDC to prevent, prepare for, and respond to coronavirus  Source: https://www.cdc.gov/cpr/readiness/funding-covid.htm | | | |
| Evaluation and testing | Confirmation of COVID-19 | | As of April 7th:  Test positive (currently 95 public health labs using CDC developed rRT-PCR test, commercial manufacturers now producing own tests) (CDC situation summary and Cases/Testing in US)  (as of March 14, 2020, public health laboratories using CDC assay are no longer required by FDA to submit samples to CDC for confirmation.)  Update as of April 14th:  Reported case counts and death counts on CDC website updated to include both confirmed and probably cases. This change was made to reflect an interim COVID-19 position statement issued by the Council for State and Territorial Epidemiologists on April 5, 2020: (https://cdn.ymaws.com/www.cste.org/resource/resmgr/2020ps/Interim-20-ID-01_COVID-19.pdf)  Confirmed case: person had confirmatory laboratory evidence  Probable case: person either  a.) met clinical criteria AND epidemiologic evidence with no confirmatory laboratory testing performed for COVID-19  OR b.) met presumptive laboratory evidence AND either clinical criteria OR epidemiologic evidence  OR c.) met vital records criteria with no confirmatory laboratory testing performed for COVID-19).    Definition of confirmed and probable: https://www.cdc.gov/coronavirus/2019-ncov/cases-updates/cases-in-us.html  Source: <https://www.cdc.gov/coronavirus/2019-ncov/covid-data/faq-surveillance.html> | | | |
| Triage protocols | Hospital admission criteria | | As of March 30th:  Patients with a mild clinical presentation (absence of viral pneumonia and hypoxia) may not initially require hospitalization, and many patients will be able to manage their illness at home. The decision to monitor a patient in the inpatient or outpatient setting should be made on a case-by-case basis. This decision will depend on the clinical presentation, requirement for supportive care, potential risk factors for severe disease, and the ability of the patients to self-isolate at home. Patients with risk factors for severe illness should be monitored closely given the possible risk of progression to severe illness in the second week after symptom onset.  Some patients with COVID-19 will have severe disease requiring hospitalization for management. Complications of severe COVID-19 include pneumonia, hypoxemic respiratory failure/ARDS, sepsis and septic shock, cardiomyopathy and arrhythmia, acute kidney injury, and complications from prolonged hospitalization including secondary bacterial infections.  Inpatient management of COVID-19 revolves around the supportive management of the most common complications of severe COVID-19: pneumonia, hypoxemic respiratory failure/ARDS, shock, multiorgan failure, and the complications associated with prolonged hospitalization including secondary nosocomial infection, thromboembolism, gastrointestinal bleeding, and critical illness polyneuropathy/myopathy.  Update as of April 3rd:  Patients with a mild clinical presentation (absence of viral pneumonia and hypoxia) may not initially require hospitalization, and many patients will be able to manage their illness at home. The decision to monitor a patient in the inpatient or outpatient setting should be made on a case-by-case basis. This decision will depend on the clinical presentation, requirement for supportive care, potential risk factors for severe disease, and the ability of the patient to self-isolate at home. Patients with risk factors for severe illness should be monitored closely given the possible risk of progression to severe illness in the second week after symptom onset.  Some patients with COVID-19 will have severe disease requiring hospitalization for management. Inpatient management revolves around the supportive management of the most common complications of severe COVID-19: pneumonia, hypoxemic respiratory failure/ARDS, sepsis and septic shock, cardiomyopathy and arrhythmia, acute kidney injury, and complications from prolonged hospitalization including secondary bacterial infections, thromboembolism, gastrointestinal bleeding, and critical illness polyneuropathy/myopathy  Source: <https://www.cdc.gov/coronavirus/2019-ncov/hcp/clinical-guidance-management-patients.html> | | | |
| Infection control | Healthcare triage isolation | | As of March 19th:  Install physical barriers (e.g., glass or plastic windows) at reception areas to limit close contact between triage personnel and potentially infectious patients.  Consider establishing triage stations outside the facility to screen patients before they enter.  Isolate the patient in an examination room with the door closed. If an examination room is not readily available ensure the patient is not allowed to wait among other patients seeking care.  Identify a separate, well-ventilated space that allows waiting patients to be separated by 6 or more feet, with easy access to respiratory hygiene supplies.  In some settings, patients might opt to wait in a personal vehicle or outside the healthcare facility where they can be contacted by mobile phone when it is their turn to be evaluated.  Additional considerations: Designate an area at the facility (e.g., an ancillary building or temporary structure) or identify a location in the area to be a “respiratory virus evaluation center” where patients with fever or respiratory symptoms can seek evaluation and care.  Update April 13th  1. Install physical barriers (e.g., glass or plastic windows) at reception areas to limit close contact between triage personnel and potentially infectious patients.  2. Consider establishing triage stations outside the facility to screen individuals before they enter.  3. Isolate patients with symptoms of COVID-19 in an examination room with the door closed. If an examination room is not readily available ensure the patient is not allowed to wait among other patients seeking care.  4. Identify a separate, well-ventilated space that allows waiting patients to be separated by 6 or more feet, with easy access to respiratory hygiene supplies.  In some settings, patients might opt to wait in a personal vehicle or outside the healthcare facility where they can be contacted by mobile phone when it is their turn to be evaluated.  Additional considerations: Designate an area at the facility (e.g., an ancillary building or temporary structure) or identify a location in the area to be a “respiratory virus evaluation center” where patients with fever patients with fever or COVID-19 symptoms can seek evaluation and care.  Source: <https://www.cdc.gov/coronavirus/2019-ncov/hcp/infection-control-recommendations.html> | | | |
|  | Visitor access to healthcare facilities | | March 19th:  -Visitors must be passively screened for symptoms of acute respiratory illness before entering the healthcare facility  -post visual alerts advising visitors not to enter facility when ill  -inform visitors about appropriate PPE and hand hygiene according to current facility visitor policy (limiting surfaces touched, use PPE while in patient's room)  -limit visitors to the most vulnerable patients (e.g. oncology and transplant wards)  -encourage use of alt. mechanisms for patient/visitor interactions (video-call applications)  -visitors should not be present during aerosol generating procedures or other specimen collection procedures  -visitors should be instructed to only visit the patient room and should not go to other locations in facility  Update April 13th  -Limit visitors to the facility to only those essential for the patient’s physical or emotional well-being and care (e.g., care partners)  -Encourage use of alternative mechanisms for patient and visitor interactions such as video-call applications on cell phones or tablets.  -Limit points of entry to the facility and visitation hours to allow screening of all potential visitors.  -Actively assess all visitors for fever and COVID-19 symptoms upon entry to the facility. If fever or COVID-19 symptoms are present, the visitor should not be allowed entry into the facility.  -Establish procedures for monitoring, managing, and training all visitors, which should include:  ---All visitors should be instructed to wear a facemask or cloth face covering at all times while in the facility, perform frequent hand hygiene, and restrict their visit to the patient’s room or other area designated by the facility.  ---Informing visitors about appropriate PPE use according to current facility visitor policy.  -If visitation to patients with COVID-19 occurs, visits should be scheduled and controlled to allow for the following:  ---Facilities should evaluate risk to the health of the visitor (e.g., visitor might have underlying illness putting them at higher risk for COVID-19) and ability to comply with precautions.  ---Facilities should provide instruction, before visitors enter patients’ rooms, on hand hygiene, limiting surfaces touched, and use of PPE according to current facility policy while in the patient’s room.  ---Visitors should not be present during AGPs or other procedures.  ----Visitors should be instructed to only visit the patient room. They should not go to other locations in the facility.  Source: <https://www.cdc.gov/coronavirus/2019-ncov/hcp/infection-control-recommendations.html> | | | |
|  |  | |  | | | |
| Themes / Sub-themes | | | **UK** | | | |
| Evaluation and testing | Screening criteria | | There is no community testing of the public for SARS2-COV as of April 9th, 2020.  COVID-19 symptoms criteria for screening and reporting.  2.1 Patients who meet the following criteria (inpatient definition)  requiring admission to hospital (a hospital practitioner has decided that admission to hospital is required with an expectation that the patient will need to stay at least one night)  and have either clinical or radiological evidence of pneumonia or acute respiratory distress syndrome or influenza like illness (fever ≥37.8°C and at least one of the following respiratory symptoms, which must be of acute onset: persistent cough (with or without sputum), hoarseness, nasal discharge or congestion, shortness of breath, sore throat, wheezing, sneezing  Note: Clinicians should consider testing inpatients with new respiratory symptoms or fever without another cause or worsening of a pre-existing respiratory condition.  2.2 Patients who meet the following criteria and are well enough to remain in the community  new continuous cough and/or high temperature  Individuals with cough or fever should now stay at home. Those staying at home are not prioritized for testing.  Clinicians should be alert to the possibility of atypical presentations in patients who are immunocompromised.  Alternative clinical diagnoses and epidemiological risk factors should be considered.  <https://www.gov.uk/government/publications/wuhan-novel-coronavirus-initial-investigation-of-possible-cases/investigation-and-initial-clinical-management-of-possible-cases-of-wuhan-novel-coronavirus-wn-cov-infection> Accessed 1^st^ April 2020 @16:45Hrs | | | |
|  | Screening center types | | Testing is confined to NHS facilities and pop up drive through sites such as Chesington World of Adventures (Theme Park) in Surrey.  These facilities are not open to the public but are reserved for NHS staff and their household member who are symptomatic only.  22nd April No change in screening criteria  <https://www.gov.uk/government/publications/wuhan-novel-coronavirus-initial-investigation-of-possible-cases/investigation-and-initial-clinical-management-of-possible-cases-of-wuhan-novel-coronavirus-wn-cov-infection> Accessed 27^th^ April 2020 @12:25Hrs | | | |
| Screening system | Outpatient appointment guidance | | Outpatient appointments are to be virtual via phone clinics or video clinics where appropriate.  COVID patients are instructed not to present at hospital (there are no outpatient clinics for COVID patients 09 April 2020). Patients with COVID symptoms are instructed to self-isolate at home.  22nd April No change  <https://www.england.nhs.uk/coronavirus/wp-content/uploads/sites/52/2020/03/C0044-Specialty-Guide-Virtual-Working-and-Coronavirus-27-March-20.pdf> Accessed 27^th^ April 2020 @12:00 Hrs | | | |
| Cost support | Cost support (testing and treatment) | | The UK Government has committed £5 Billion to address coronavirus with a pledge to fund the NHS with whatever it needs. **22nd April update** All healthcare expenses relating to COVID19 are covered by the NHS as they have been since the beginning of the pandemic.  <https://www.nhs.uk/using-the-nhs/nhs-services/visiting-or-moving-to-england/visitors-who-do-not-need-pay-for-nhs-treatment/> Accessed 27^th^ April 2020 @12:39Hrs | | | |
| Evaluation and testing | Confirmation of COVID-19 | | A COVID19 positive test post swabbing (PCR) is required to be considered. Clinical symptoms and CxR findings alone are not enough.  <https://www.england.nhs.uk/coronavirus/wp-content/uploads/sites/52/2020/03/clinical-management-of-persons-admitted-to-hospita-v1-19-march-2020.pdf> | | | |
| Triage protocols | Hospital admission criteria | | Only patients who meet the following criteria are admitted 2.1 Patients who meet the following criteria are only tested once they are on a ward as an inpatient. Currently the NHS Trusts are still trying to work to the National four hour target to be admitted from the ED. (This depends on availability of beds, delays in patients not being stable enough to transfer, a duty of Pt and internal communication issues). (inpatient definition)  • requiring admission to hospital (a hospital practitioner has decided that admission to hospital is required with an expectation that the patient will need to stay at least one night)  and  • have either clinical or radiological evidence of pneumonia  or  • acute respiratory distress syndrome  or  • influenza like illness (fever ≥37.8°C and at least one of the following respiratory symptoms, which must be of acute onset: persistent cough (with or without sputum), hoarseness, nasal discharge or congestion, shortness of breath, sore throat, wheezing, sneezing  Note: Clinicians should consider testing inpatients with new respiratory symptoms or fever without another cause or worsening of a pre-existing respiratory condition. (Patients are not swabbed prior to admission and a bed on a ward, swabbing is not performed in the ED.)  <https://www.england.nhs.uk/coronavirus/wp-content/uploads/sites/52/2020/03/clinical-management-of-persons-admitted-to-hospita-v1-19-march-2020.pdf> Accessed 1^st^ April 2020 @16:00 Hrs | | | |
| Infection control | Healthcare triage isolation | | Clinicians should:  implement infection prevention and control measures whilst awaiting test results, including isolation and cohorting of patients in line with your Trust seasonal influenza operational plan  assess individuals in a single occupancy room  wear personal protective equipment (PPE) - as a minimum, this should be a fluid resistant surgical mask, single use disposable apron and gloves and eye protection if blood and or body fluid contamination to the eyes or face is anticipated. If a patient meeting the case definition undergoes an aerosol generating procedure, then a FFP3 respirator, long-sleeved disposable fluid-repellent gown, gloves, and eye protection must be worn; refer to infection prevention and control (IPC) guidance and PPE guidance  ask the patient to wear a fluid-resistant (Type IIR) surgical face mask (FRSM) if they are in a clinical or communal area or are being transported if the patient can tolerate it. The aim of this is to minimize the dispersal of respiratory secretions, reduce both direct transmission risk and environmental contamination. A FRSM should not be worn by patients if there is potential for their clinical care to be compromised (for example, when receiving oxygen therapy via a mask). An FRSM can be worn until damp or uncomfortable. 3.1 Isolation  Ensure the patient is placed in respiratory isolation or within a specified cohort bay and the PPE described in the infection prevention and control guidance is worn by any person entering the room.  Ensure that the patient, potentially contaminated areas, and waste are managed as per the infection prevention and control guidance.  <https://www.gov.uk/government/publications/wuhan-novel-coronavirus-initial-investigation-of-possible-cases/investigation-and-initial-clinical-management-of-possible-cases-of-wuhan-novel-coronavirus-wn-cov-infection> | | | |
|  | Visitor access to healthcare facilities | | Our preference at this point is that we have no visitors coming to our hospitals.  We will, however, consider visitors on compassionate grounds for seriously ill patients or those receiving end-of-life care only in agreement with each individual ward. 22 April 2020 Visitor access to COVID-19 patients varies from NHS Trust to NHS Trust. The latest NHS England guidance states that hospital visiting is "suspended with immediate effect and until further notice", but it lists several exceptions.  It says in certain circumstances one visitor - who must be an immediate family member or carer - is allowed to visit a hospital patient.  The circumstances include:  If the patient is receiving end-of-life care, If the visitor is the birthing partner of a woman in labor, If the visitor is a parent or "appropriate adult" visiting a child patient, If the visitor is supporting someone with a mental health issue such as dementia, a learning disability or autism, where not being present would cause the patient to be distressed, The guidance applies to all inpatient, diagnostic and outpatient areas.  <https://www.england.nhs.uk/coronavirus/wp-content/uploads/sites/52/2020/03/C0030_Visitor-Guidance_8-April-2020.pdf> | | | |
|  |  | |  | | | |
| Themes / Sub-themes | | | **Haiti** | | | |
|  |  |  | Verbatum | | | Translation |
| Evaluation and testing | | Screening criteria | Toute personne avec un antécédent de fièvre ou une fièvre supérieure ou égale à 38 de- grés Celsius et de la toux avec une apparition dans les 10 der- niers jours avec ou sans critères d’hospitalisation. Antécédents de voyage dans des pays (zones) affectés au CO- VID-19 durant les 14 jours pré- cédant l’apparition des symp- tômes de grippe  Ou A été en contact avec un cas confirmé de COVID-19. Au niveau des institutions tout patient avec une infection aigue des voies respiratoires su- périeures (IVRS) d’au moins cinq jours, ne répondant pas au traitement usuel, dont l’état de santé se détériore avec tendance à développer une pneumonie ou broncho-pneumonie et dont les antécédents révèlent qu’il a été en contact avec une personne malade ou qu’il vient d’un pays où le COVID-19 a été confirmé sera considérée comme suspect. Chez ces patients suspects deux échantillons seront pris: un prélèvement nasopharyngé et oro-pharyngé. Updated as of 4/20/20: Toute personne présentant une fièvre supérieure ou égale à 38 degrés Celsius, ou antécédent de fièvre, acompagnée de toux avec ou sans dificultés respiratoires, céphalée, courbatureset/ouéventuelement ayant eu contact avec un cas confirmé de COVID-19.  Ou Toute personne présentant une altération subite du gout (agueusie) oudel’odorat (anosmie)  Ou Toute personne chez qui un personnel de santé (Médecin ou infirmière...) pose undiagnostic de COVID-19.  Ou Toute personne en provenance d’une zone à risque avec des symptômes compatibles à la COVID-19 | | | Anyone with a history of fever or fever greater than or equal to 38°C and cough with onset within the last 10 days with or without criteria for hospitalization. Travel history in countries (zones) assigned to COVID 19 during the 14 days preceding the onset of flu symptoms  Or Has been in contact with a confirmed case of COVID-19 with a history of fever or have a fever greater than 38°C and a cough within the last 10 days. At the institutional level, any patient with an acute upper respiratory infection (URTI) of at least five days, who does not respond to usual treatment, whose state of health deteriorates with a tendency to develop pneumonia or broncho- pneumonia and whose history indicates that he has been in contact with a sick person or that he comes from a country where COVID-19 has been confirmed will be considered suspicious. In these suspect patients two samples will be taken: a nasopharyngeal and an oropharyngeal sample. Updated as of 4/20/20: Anyone with fever greater than or equal to 38 degrees Celsius, or history of fever, accompanied by cough with or without respiratory difficulties, headache, body aches and / or possibly having had contact with a confirmed COVID-19. Or Anyone with sudden changes in taste (ageusia) or smell (anosmia). Or Anyone with a health staff (doctor or nurse, etc.) diagnosed with COVID-19. Or Anyone from a risk area with symptoms compatible with COVID-19 |
|  |  | Screening center types | Not indicated | | | The government guideline is not indicated this information, however, acceding to other resources, some hospital does the screening test. |
| Screening system | | Outpatient appointment guidance | Not indicated | | | Not indicated (or unavailable) for the general population. There is some guidance for those with HIV/AIDS. Haiti has a high number of individuals suffering from HIV/AIDS |
| Cost support | | Cost support (testing and treatment) | Not indicated | | | World Bank's Board of Executive Directors approved a US$20 million grant for the Haiti COVID-19 Response Project. Other international and private organizations have donated money or promised to do so. The government in Haiti estimated that more than 37 million will be needed to respond to COVID |
| Evaluation and testing | | Confirmation of COVID-19 | Un cas confirmé est un cas suspect dont l’examen de laboratoire par PCR (effectué sur prélèvement naso-pharyngé et oro-pharyngé) ou l’examen sérologique a mis en évidence la présence d’anticorps dirigé contre le virus ou l’identification du Virus SRAS-COV-2 | | | A confirmed case is a suspect case whose laboratory examination by PCR (carried out on nasopharyngeal and oropharyngeal specimen) or serological examination revealed the presence of antibodies directed against the virus or the identification of SARS-COV-2 virus |
| Triage protocols | | Hospital admission criteria | Not indicated | | | This information is not indicated anywhere on the government website. Keep in mind that more than 60% of health care in Haiti is provided by private actors so the government website may not have this information since private actors may establish different criteria. |
| Infection control | | Healthcare triage isolation | Deux groupes de centres d’isolement sont prévus pour la prise en charge Clinique : Des centres d’isolement de niveau 1, pour la PEC des cas les plus simples (59 centres de niveau 1, totalisant une capacité installée de 577 lits seront installés dans les 10 dé- partements sanitaires du pays). Des centres d’isolement de niveau 2 pour la PEC des cas compliqués. | | | Two groups of isolation centers are planned for clinical care: Level 1 isolation centers, to support the simplest cases (59 level 1 centers, totaling an installed capacity of 577 beds will be installed in the 10 health departments in the country). Level 2 isolation centers for the support of complicated cases. |
|  |  | Visitor access to healthcare facilities | Les visites des malades seront réduites au minimum possible et se feront avec un port de masque qui est obligatoire pour tout visiteur. | | | Patient visits will be reduced to the minimum possible and will be done with a mask that is mandatory for all visitors. |
|  |  |  |  | | | |

| Themes / Sub-themes | | South Korea | |
| --- | --- | --- | --- |
|  |  | Verbatum | Translation |
| Prevent getting sick | Prevent getting sick | 코로나바이러스감염증- 19 예방  꼭 기억해야 할 행동수칙  국민 예방수칙  흐는 물에 비누로 30초이상 꼼꼼하게 손씻기  기침이나 재채기할 때 옷소매로 입과 코 가리기  씻지 않은 손으로 눈.코.입 만지지 않기  발열, 호흡기 증상자와의 접촉 피하기  주위환경을 자주 소독하고 환기하기  의료기관 방문 시 마스크 꼭 착용하기 (특히 임신부, 65세 이상, 만성질환자 외출 시 꼭 준수)  사람 많은 곳 방문 자제하기 (특히 임신부, 65세 이상, 만성질환자 외출 시 꼭 준수) -질병관리본부, 02.25.2020  Update version on March 25, 2020 is written in English, original text displayed in the translation section.  Update version on April 8, 2020 is written in English, original text displayed in the translation section. | COVID-19 Guideline  The general public  Wash your hands thoroughly with soap and running water  Cover your mouth and nose with your elbow when coughing or sneezing  Do not touch your eyes, nose, or mouth with unwashed hands  Avoid coming in contact with people having fever or respiratory symptoms  Wear a facemask when visiting a health facility (be careful especially people who pregnant women, over 65years old, people with chronic disease)  Avoid visiting a crowed place  < Source: Response Guidelines for Coronavirus-19 (edition 7-4), Central Disease Control Headquarters, as of February 25, 2020>  **Updated: March 25, 2020**  - Delay or cancel nonessential gathering, dining-out, event, travel, etc.  * Delay or cancel events or gatherings especially providing meals as many international cases were reported to get infected due to eating together.  - Refrain from going out except for buying necessities, visiting a doctor, commuting to/from work  - Avoid handshakes and physical contact and keep a 2-meter distance  - Follow personal hygiene practices such as hand washing, cough etiquette  - Disinfect and ventilate surroundings every day  < Source: Response Guidelines for Coronavirus-19 (edition 7-4), Central Disease Control Headquarters, as of March 25, 2020>  **Updated: April 8, 2020**  - All Koreans and long-stay Foreigners should mandatorily install the “Self-quarantine Safety Protection App” by the Ministry of the Interior and Safety and abide by the guidelines for self-quarantined persons including conducting self-diagnosis for a period of 14 days.  < Source: Coronavirus Disease-19, Central Disease Control Headquarters, as of April 8, 2020> |
| If you are sick | What to do if you are sick | 코로나바이러스감염증- 19 예방  꼭 기억해야 할 행동수칙  유증상자 예방수칙  등교나 출근을 하지 않고 외출 자제하기  3-4일 경고를 관찰하며 집에서 충분히 휴식하기  38 °C 이상 고열이 지속되거나 증상이 심해질 경우 콜센터 (1339, 지역번호+120) 관할 보건소 문의 및 선별진료소 우선방문 후 진료받기  국내 코로나19 유행지역에서는 외출, 타지역 방문을 자제하고 격리자는 의료인, 방역당국의 지시 철저히 따르기  진료의료진에게 해외여행력 및 호흡기 증상자와의 접촉여부 알리기  의료기관 방문 시 마스트 착용 및 자차 이용하기 -  자가격리대상자 생활수칙  1. 감염 전파 방지 위해 격리장소 외 외출 금지  2. 독립된 공간에서 혼자 생활하기  - 방문은 닫고 창문을 열어 자주 환기시키기  - 식사는 혼자서 하기  - 가능한 혼자만 사용할 수 있는 화장실과 세면대가 있는 공간 사용하기  (불가피하게 공용으로 사용한다면 사용 후 락스 등 가정용 소독제로 소독하기)  3. 진료 등 외출이 불가피한 경우 반드시 관할 보건소에 먼저 연락하기  4. 가족 또는 동거인과 대화 등 접촉 피하기 (불가피할 경우 마스크를 쓴 채 서로 2m 이상 거리 두기)  5. 수건, 식기류 등 생활물품 분류해 개인적으로 사용하기  (의복 및 침구류 단독세탁, 식기류는 별도 분리해 씻기)  6. 손씻기, 기침예절 준수, 마스크 착용 등 기본 감염예방수칙 철저히 지키기  <제공: 질병관리본부, 02.03.2020> | COVID-19 Guideline  Person with symptoms  Do not to school or work avoid outdoor activities  Take a rest at home and monitor the symptoms for 3-4days  Consult with KCDC Call center at 1339, a local code+120 or a local health center (visit a triage health center when fever (38 °C) continues, or other symptoms get worse  Avoid visiting other regions or having outdoor activities and (person in isolation) please follow guidance provided by physicians and public health authority. (COVID-19 Outbreak reported regions in Korea)  Inform your healthcare provider of a travel history and contact history with persons with respiratory symptoms  Use a personal vehicle and wear a facemask when visiting a health facility  Person with quarantine  Stay in quarantine place to prevent the spread of infection  Separate self from others as much as possible  Close door and open window for ventilation  Eat in own room if possible  Use a separate bathroom if available (If it is necessary to use a common bathroom, disinfect after use)  Avoid contact with family or others (if necessary, keep 2-meters and wearing a mask)  Avoid sharing personal household items (dishes, drinking glasses, utensils, towels, bedding) and wash used items thoroughly after use  Cover coughs and sneezes, disposing of used tissues in lined trash can and washing hands immediately afterward, wear a cloth covering over nose  < Source: Coronavirus Disease-19, Central Disease Control Headquarters, as of February 03, 2020> |
|  | Threshold to contact healthcare provider | 38 °C 이상 고열이 지속되거나 증상이 심해질 경우 콜센터 (1339, 지역번호+120) 관할 보건소 문의 및 선별진료소 우선방문 후 진료받기  <제공: 질병관리본부, 02.03.2020> | Consult with KCDC Call center at 1339, a local code+120 or a local health center (visit a triage health center when fever (38 °C) continues, or other symptoms get worse  < Source: Coronavirus Disease-19, Central Disease Control Headquarters, as of February 03, 2020> |
|  | Transport to healthcare facilities | 자차 혹은 119 이용  <제공: 질병관리본부, 02.03.2020> | Personal vehicle with a facemask or call 119  < Source: Coronavirus Disease-19, Central Disease Control Headquarters, as of February 03, 2020> |
| Themes / Sub-themes | | **Brazil** | |
|  |  | Verbatum | Translation |
| Prevent getting sick | Prevent getting sick | Lave com frequência as mãos até a altura dos punhos, com água e sabão, ou então higienize com álcool em gel 70%. Mantenha os ambientes limpos e bem ventilados. Não compartilhe objetos de uso pessoal, como talheres, toalhas, pratos e copos. Ao tossir ou espirrar, cubra nariz e boca com lenço ou com o braço, e não com as mãos. Higienize com frequência o celular e os brinquedos das crianças. Evite tocar olhos, nariz e boca com as mãos não lavadas. Ao tocar, lave sempre as mãos como já indicado. Mantenha uma distância mínima de cerca de 2 metros de qualquer pessoa tossindo ou espirrando. Evite abraços, beijos e apertos de mãos. Adote um comportamento amigável sem contato físico, mas sempre com um sorriso no rosto. Se estiver doente, evite contato físico com outras pessoas, principalmente idosos e doentes crônicos, e fique em casa até melhorar. Durma bem e tenha uma alimentação saudável. (Source: Cartaz geral) | Wash your hands frequently up to the wrists with soap and water, or clean with 70%  alcohol gel.  When coughing or sneezing, cover your nose and mouth with a tissue or your arm,  not with your hands.  Avoid touching your eyes, nose, and mouth with your unwashed hands. When  touching, always wash hands as indicated  Keep a minimum distance of about 2 meters from anyone coughing or sneezing  Avoid hugs, kisses, and handshakes. Adopt friendly behavior without physical  contact, but always with a smile on his face.  Frequently sanitize cell phones and children's toys.  Do not share personal items such as cutlery, towels, plates and glasses  Keep environments clean and well ventilated.  If you are sick, avoid physical contact with other people, especially the elderly and  chronically ill and stay home until you get better.  Sleep well and eat healthy. |
| If you are sick | What to do if you are sick | Orientações para isolamento domiciliar: A distância mínima entre o paciente e os demais moradores é de 1 metro. No quarto usado para o isolamento, mantenha as janelas abertas para circulação do ar. A porta deve estar fechada durante todo o isolamento. Limpe a maçaneta frequentemente com álcool 70% ou água sanitária.  ATENÇÃO: EM CASAS COM APENAS UM QUARTO, OS DEMAIS MORADORES DEVEM DORMIR NA SALA, LONGE DO PACIENTE INFECTADO.  Itens que precisam ser separados: O lixo produzido pelo paciente contaminado precisa ser separado e descartado. Toalhas de banho, garfos, facas, colheres, copos e outros objetos usados pelo paciente. Sofás e cadeiras também não podem ser compartilhados. Os móveis da casa precisam ser limpos frequentemente com água sanitária ou álcool 70%.  Condutas para a pessoa contaminada:Utilize máscara o tempo todo. Se for preciso cozinhar, use máscara de proteção, cobrindo boca e nariz todo o tempo. Depois de usar o banheiro, nunca deixe de lavar as mãos com água e sabão e sempre limpe vaso, pia e demais superfícies com álcool ou água sanitária para desinfecção do ambiente.  Condutas de todos os moradores: Se uma pessoa da casa tiver diagnóstico positivo, todos os moradores ficam em isolamento por 14 dias também. Caso outro familiar da casa também inicie os sintomas leves, ele deve reiniciar o isolamento de 14 dias. Se os sintomas forem graves, como dificuldade para respirar, ele deve procurar orientação médica. (Source: cartilha- siolamento domiciliar) | Guidelines for home isolation: The minimum distance between the patient and other residents is 1 meter. In the room used for insolation, keep the windows open for air circulation. The door must be closed for the duration of the insolation. Clean the handle frequently with 70% alcohol or bleach.  ATTENTION: IN HOUSES WITH ONLY ONE ROOM, OTHER RESIDENTS MUST SLEEP IN THE LIVING ROOM, AWAY FROM THE INFECTED PATIENT.  Items that need to be separated: The waste produced by the contaminated patient needs to be separated and disposed. Bath towels, forks, knives, spoons, glasses and other objects used by the patient. Sofas and chairs cannot be shared either. Household furniture needs to be cleaned frequently with bleach or 70% alcohol.  Conduct for the infected person: Wear a mask at all times. If cooking is necessary, wear a face mask, covering your mouth and nose at all times. After using the bathroom, never [skip] washing your hands with soap and water and always clean the toilet, sink and other surfaces with alcohol or bleach to disinfect the environment.  Conduct of all residents: If a person in the household has a positive diagnosis, all residents are in isolation for 14 days as well. If another member of the household also starts with mild symptoms, they should restart isolation for 14 days. If the symptoms are severe, such as difficulty breathing, he should seek medical advice. |
|  | Threshold to contact healthcare provider | SÓ PROCURE UM HOSPITAL DE REFERÊNCIA  SE ESTIVER COM FALTA DE AR | ONLY LOOK FOR A REFERENCE HOSPITAL  IF YOU ARE OUT OF AIR [BREATHLESS] |
|  | Transport to healthcare facilities | Not specified | Not specified |
| Themes / Sub-themes | | **China** | |
|  |  | Verbatum | Translation |
| Prevent getting sick | Prevent getting sick | (1)尽量减少外出活动  1)避免去疾病止在流行的地区。  2)建议疾病流行期间减少走亲访友和聚餐,尽量在家休息。  3)减少到人员密集的公共场所活动,尤其是空气流动性差的地方,例如:公共浴池、温泉、影院，网吧、KT、商场、车站、机场、码头、展览馆等。  (2)个人防护和手卫生  1)建议外出佩戴口罩。外出前往公共场所、前往非发热门诊就医、乘坐公共交通具时,佩戴一次性使用医用口罩;如去发热门诊就医时,可佩戴医用外科罩。  2)保持手卫生。减少接触公共场所的公用物品和部位;从公共场所返回、咳嗽手捂之后、饭前便后用洗手液(肥皂)流水洗手,或者使用含酒精成分的免洗手消毒剂;如无洗手或使用免洗手消毒剂条件。可戴手套(不露手指的手套均可,同时注意保持手套干燥)。脱掉手套后,需要彻底清洗手部。不确定手是否清洁时,避免用于接触口、、眼;打喷嚏或咳嗽时,用手肘衣服遮住口、鼻。  (3)健康监测与就医  1)主动做好个人与家庭成员的健康监测,自觉发热时要主动测量体温。家中有小孩的,要早晚为其测量体温。  2)若出现可疑症状,应主动戴上口罩及时就近就医。若出现新型冠状病毒感染可疑症状(包括发热、乏力、咳嗽、咽痛、胸闷、呼吸困难、恶心呕吐腹泻、结膜炎、肌肉酸痈等),应根据病情,及时到医疗机构就诊。尽量避免乘坐地铁、公共汽车等交通工具,避免前往人员密集的场所。  (4)保持良好卫生和健康习惯  1)居室勤开窗,经常通风  2)家庭成员不共用毛巾,保持家居、餐具清洁,勤晒衣被。  3)不随地吐痰,口鼻分泌物用纸巾包好,弃置于有盖垃圾桶内。  4)注意营养,适度运动。  5)不要接触、购买利食用野生动物(即野味);尽量避免前社售实活体动物(离类、海广品、野生动物等)的市场。  6)家庭备置体温计、一次性使用医用门罩、家庭消毒用品等物资  《中国疾病预防控制中心》 | 1) Minimize outdoor activities  1. Avoid going to epidemic area  2. Avoid visiting people and party event, stay at home.  3. Reduce the time of visiting public area which is crowded and has poor ventilation, such as public bathroom, hot spring, theater, KTV, shopping mall, train station, airport, wharf, gallery, etc.  2) Individual protection and hand hygiene  1. Wear mask when go outside. Wear disposable medical facemask when you head to public area, taking public transportations. Wear surgical mask when you visit the fever clinic in hospital.  2. Keep good hand hygiene. Reduce frequency of touching items in public area. Use alcohol-based hand sanitizer or soap to clean hands before dinning, after using restroom, after covering cough and sneeze, after visiting public places. Wear gloves are recommended.  Clean hands after removing gloves. Avoid touch face with hands when you uncertain about hands’ cleanness. Use elbow to cover mouth and nose when you sneeze and cough.  3) Health monitoring and visit doctor  1. Monitor health condition for each family member. Take a body temperature measurement when you have fever. Measure body temperature twice per day for kids at home.  2. Immediately wear mask and go to see doctor if suspicious symptoms occur. If any Covid-19 related symptoms occur (Fever, fatigue, cough, sore throat, chest tightness, dyspnea, nausea, vomiting, diarrhea, conjunctivitis, muscle soreness, etc.), go visit medical care institution in time. Avoid taking public transportations and do not go to crowded places.  4) Maintain good hygiene and healthy habits  1. Keep the window open as much as possible to maintain a good ventilation.  2. Do not share towel with family, use clean tableware, use sunlight to sanitize cloth and beddings.  3. Do not spit casually. Cover it up by tissue and toss it to trashcan.  4. Balanced diet, take exercise regularly.  5. Do not touch or purchase wild animals. Avoid going to market where sell live creatures.  6. Store the thermometer, disposable face mask and sanitizers at home.  <Chinese Center for Disease Control and Prevention> |
| If you are sick | What to do if you are sick | (1)若出现新型冠状病毒肺炎可疑症状(包括发热、干咳、乏力、鼻塞、流涕、咽痛、肌痛和腹泻等症状),应尽快前往定点医疗机构进行采样和实验室检测,并按照要求进行隔离医学观察。  (2)避免乘坐地铁、公共汽车等公共交通工具,避免前往人员密集的场所。  (3)就诊时应主动告诉医生自己的相关疾病流行地区的旅行居住史,以及发病后接触过什么人,配合医生开展相关调查。  (4)如需开展居家隔离医学观察,则家人均应佩戴一次性使用医用口罩或医用外科口罩。可疑症状者需尽量避免与家庭成员接触,如需共处一室,建议保持距离,相隔1米以上。  (5)若家庭中有人被诊断为新型冠状病毒肺炎,其他家庭成员如果经判定为密切接触者,应接受14天隔离医学观察。  (6)如家庭成员确诊为新型冠状病毒肺炎,则其住所、生活用品、衣物、寝具、餐具等,均需要终末消毒后才能使用。  《中国疾病预防控制中心》 | 1. If you have Covid-19 related suspicious symptoms (include fever, fatigue, dry cough, sore throat, dyspnea, diarrhea, conjunctivitis, muscle soreness, etc.), immediately go to the designated medical care institution for having specimen collection and lab analysis and follow the quarantine protocols as request.  2. Avoid using public transportations and do not go to crowded places.  3. Voluntarily report your travel history to epidemic area, contact list when you visit doctor, cooperate with your doctor to do relevant researches.  4. If a home quarantine initiated, all family members should wear the disposable medical face mask. Person with suspicious symptoms has to avoid physical contact with other people. Always keep 1-meter distance with others.  5. If one person has confirmed infection of Covid-19, all other family members are classified as close contacts, require taking a 14 days quarantine.  6. If one person has confirmed infection of Covid-19, its residence, supplies, cloth, beddings, tableware and other belongings have to take the procedure of final disinfection, for future reuse.  <Chinese Center for Disease Control and Prevention> |
|  | Threshold to contact healthcare provider | 对于健康的成年人而言，如果出现发热，呼吸道感染，急性消化道症状，在原有症状对症治疗后不能缓解或症状加重，或出现其他可疑症状如呼吸困难，腹泻等，或其他家庭成员也出现新型冠状病毒感染的可疑症状时，应该及时就医。但糖尿病，免疫功能缺陷，肝肾功能不全，心脑血管疾病等基础性疾病患者，老年人，孕妇等是新冠病毒感染重症高危人群，出现可疑症状后需要立即就医。  《中国疾病预防控制中心》 | In terms of the healthy adults, visit doctors when you have fever, respiratory infections, acute digestive tract symptoms and when the symptoms do not decrease or aggravate after symptomatic treatment; or if other family members start having Covid-19 related suspicious symptoms.  But for people who have diabetes, immunodeficiency, hepatic and renal insufficiency, cardiovascular and cerebrovascular diseases or other underlying diseases, plus the seniors and pregnant women are high-risk groups for severe condition of Covid-19 infection. They must see doctors immediately after the occurrence of suspicious symptoms.  <Chinese Center for Disease Control and Prevention> |
|  | Transport to healthcare facilities | 就医途中应佩戴医用外科口罩，避免乘坐公共交通工具前往  《中国疾病预防控制中心》 | People must wear surgical mask on the way to hospital; Avoid taking the public transportation to go to hospital.  <Chinese Center for Disease Control and Prevention> |
| Themes / Sub-themes | | **U.S.A.** | |
| Prevent getting sick | Prevent getting sick | -The best way to prevent illness is to avoid being exposed to this virus  -Know how it spreads: The virus is thought to spread mainly from person-to-person: Between people who are in close contact with one another (within about 6 feet) and through respiratory droplets produced when an infected person coughs or sneezes.  -Clean your hands often: Wash your hands often with soap and water for at least 20 seconds especially after you have been in a public place, or after blowing your nose, coughing, or sneezing.  If soap and water are not readily available, use a hand sanitizer that contains at least 60% alcohol. Cover all surfaces of your hands and rub them together until they feel dry. Avoid touching your eyes, nose, and mouth with unwashed hands.  -Avoid close contact: Avoid close contact with people who are sick. Put distance between yourself and other people if COVID-19 is spreading in your community.  -Stay home if you are sick, except to get medical care.  -Cover coughs and sneezes: cover coughs and sneezes with a tissue or use the inside of your elbow. Throw used tissues in the trash. Immediately wash your hands or use hand sanitizer afterward.  -Wear a facemask if you are sick. If you are sick you should wear a facemask when you are around other people (e.g. sharing a room or vehicle and before you enter a healthcare provider's office. If you are NOT sick: You do not need to wear a facemask unless you are caring for someone who is sick (and they are not able to wear a facemask). Facemasks may be in short supply and they should be saved for caregivers.  -Clean and disinfect: Clean AND disinfect frequently touched surfaces (includes tables, doorknobs, light switches, countertops, handles, desks, phones, keyboards, toilets, faucets, and sinks.). If surfaces are dirty, clean them: Use detergent or soap and water prior to disinfection.  Updates as of April 4th:  -Know how it spreads: The virus is thought to spread mainly from person-to-person: Between people who are in close contact with one another (within about 6 feet) and through respiratory droplets produced when an infected person coughs, sneezes, or talks. Some recent studies have suggested that COVID-19 may be spread by people who are not showing symptoms.  -Avoid close contact: Stay at home as much as possible. Put distance between yourself and other people. Remember that some people without symptoms may be able to spread the virus.  -Cover your mouth and nose with a cloth face cover when around others: You could spread COVID-19 to others even if you do not feel sick. Everyone should wear a cloth face cover when they must go out in public, for example to the grocery store or to pick up other necessities. Do NOT use a facemask meant for a healthcare worker. Continue to keep about 6 feet between yourself and others. The cloth face cover is not a substitute for social distancing.  -Cover coughs and sneezes: If you are in a private setting and do not have on your cloth face covering, remember to always cover your mouth and nose with a tissue when you cough or sneeze, or use the inside of your elbow. Throw used tissues in the trash. Immediately wash hands afterward or use hand sanitizer.  -Update on April 13, 2020  -Clean and disinfect: If surfaces are dirty, clean them: Use detergent or soap and water prior to disinfection. Then, use a household disinfectant. Most common EPA-registered household disinfectant will work. | |
| If you are sick | What to do if you are sick | As of April 5th:  -Stay home except to get medical care: Most people with COVID-19 have mild illness and are able to recover at home without medical care.  Do not leave your home, except to get medical care. Do not visit public areas.  Avoid public transportation: Avoid using public transportation, ridesharing, or taxis.  -Separate yourself from other people and pets in your home, this is known as home  isolation: Stay away from others: As much as possible, you stay away from others. You should stay in a specific "sick room" if possible, and away from other people and pets in your home. Use a separate bathroom if available.  -Call ahead before visiting your doctor  -If you are sick wear a cloth covering over your nose and mouth: You should wear a cloth face covering, over your nose and mouth if you must be around other people even at home. During the COVID-19 pandemic, medical grade facemasks are reserved for healthcare workers and some first responders. You may need to improvise a cloth face covering using a scarf or bandana  -Cover your coughs and sneezes: cover your mouth and nose with a tissue when you cough or sneeze, dispose of used tissues in a lined trash can. Wash hands immediately afterward with soap and water for at least 20 seconds. If soap and water are not available, clean your hands with an alcohol-based hand sanitizer that contain at least 60% alcohol.  -Clean your hands often (soap and water for at least 20 seconds or hand sanitizer with at least 60% alcohol, covering all surfaces of your hands and rubbing them together until they feel dry). Avoid touching your eyes, nose, and mouth with unwashed hands.  -Avoid sharing personal household items: Do not share dishes, drinking glasses, cups, eating utensils, towels, or bedding with other people in your home. Wash items thoroughly after use (with soap and water or put in the dishwasher).  -Clean all "high-touch" surfaces everyday: Clean high-touch surfaces in your isolation area (“sick room” and bathroom) every day; let a caregiver clean and disinfect high-touch surfaces in other areas of the home. If a caregiver or other person needs to clean and disinfect a sick person’s bedroom or bathroom, they should do so on an as-needed basis. The caregiver/other person should wear a mask and wait as long as possible after the sick person has used the bathroom.  Clean and disinfect areas that may have blood, stool, or body fluids on them  -Monitor your symptoms: Trouble breathing is a more serious symptom that means you should get medical attention.  -People with COVID-19 who have stayed home (home isolated) can stop home isolation under the following conditions:  --if will not have a test to determine if still contagious, can leave home after the following three things have happened: have had no fever for at least 72 hours without the use of medicine that reduces fevers, other symptoms have improved (ex: cough or shortness of breath), and at least 7 days have passed since symptoms first appeared.  --if you will be tested to determine if still contagious, can leave home after the following three things have happened: you no longer have a fever (without the use of medicine that reduces fevers), other symptoms have improved, and you receive 2 negative tests in a row, 24 hours apart.  -In all cases, follow the guidance of your healthcare provider and local health department. The decision to stop home isolation should be made in consultation with your healthcare provider and state and local health departments.  Update April 9th  -Stay home except to get medical care: Take care of yourself. Get rest and stay hydrated.  -If you are sick wear a cloth covering over your nose and mouth: To prevent the spread of COVID-19, wear a cloth face covering, over your nose and mouth if you must be around other people even at home.  You don’t need to wear the cloth face covering if you are alone. If you cannot put on a cloth face covering (because of trouble breathing for example), cover your coughs and sneezes in some other way. Try to stay at least 6 feet away from other people. This will help protect the people around you.  Update April 16th  -Separate yourself from other people and pets in your home: As much as possible, stay in a specific room and away from other people and pets in your home. Also, you should use a separate bathroom, if available. If you need to be around other people or animals in or outside of the home, wear a cloth face covering.  -If you are sick wear a cloth covering over your nose and mouth: You should wear a cloth face covering, over your nose and mouth if you must be around other people or animals, including pets (even at home). | |
|  | Threshold to contact healthcare provider | As of April 5th  -If you develop emergency warning signs for COVID-19 get medical attention immediately. Emergency warning signs  include*:  -Trouble breathing  -Persistent pain or pressure in the chest  -New confusion or inability to arouse  -Bluish lips or face  *This list is not all inclusive. Please consult your medical provider for any other symptoms that are severe or concerning.  Update April 16th  -New confusion or not able to be woken  https://www.cdc.gov/coronavirus/2019-ncov/if-you-are-sick/steps-when-sick.html | |
|  | Transport to healthcare facilities | As of April 5th  -Personal vehicle  -Avoid public transport, ridesharing, or taxis.  -If called 911, put on a cloth face covering that covers nose and mouth before medical help arrives (would be transported via ambulance)  Update April 9th  -If called 911, put on a face mask before medical help arrives (would be transported via ambulance)  Update April 16th  -If called 911, put on a cloth face covering before medical help arrives (would be transported via ambulance) | |
| Themes / Sub-themes | | **UK** | |
| Prevent getting sick | Prevent getting sick | 1) Keep your hands clean.  Wash your hands lots of times during the day: use soap and water ; use hand sanitizer (gel) if there is no soap and water When you are out, make sure you wash your hands when: • you come home • you visit other places • before you touch food  Do not touch your eyes, nose or mouth  Always cough and sneeze into a tissue. Then throw the tissue away and wash your hands.  2) Stay at home  Only go outside for food, health reasons or work (but only if you cannot work from home)  If you go out, always stay 2 meters (6ft) away from other people  Wash your hands as soon as you get home (For 20 seconds)  Do not meet others, even friends or family. You can spread the virus even if you don’t have symptoms. | |
| If you are sick | What to do if you are sick | Stay at home and don’t meet up with other people for 7 days if you have Coronavirus symptoms.  These are:  • a new cough, and you keep coughing or • a high temperature    After 7 days, if you feel better, you can start your usual routine again. | |
|  | Threshold to contact healthcare provider | Only use health services when it is important to do so.    If you:    • are staying at home and start to feel much more ill or • have stayed at home for 7 days and still have symptoms of Coronavirus    then:    • get advice from NHS 111 online • if you need to speak to someone call 111 • you should not go to a GP surgery, pharmacy, or hospital as you could pass Coronavirus to others    Only dial 999 or go to Accident and Emergency if there is an emergency. | |
|  | Transport to healthcare facilities | By Ambulance which will be arranged following contact with www.111.nhs.uk/covid-19 or the 111 NHS telephone service | |
| Themes / Sub-themes | | **Haiti** | |
|  |  | Verbatum | Translation |
| Prevent getting sick | Prevent getting sick | Toujou lave men ou ak dlo ak savon, oswa sevi ak pwodui ki fèt pousa. Touse oswa estènen nan koud bra nou oswa nan mouchwa ki ka sèvi yon sel fwa. Sonje toujou lave men nou anvan nou manyen bouch nou, je nou ak nen nou. Pwoteje tèt ou sizok ou dwe rete pre osinon kole ak yon moun ki mal pou respire, kap touse oswa kap estènen. Les voyageurs de retour des zones affectées ou qui ont eu un contact avec un cas confirmé dans les 14 jours précédant leur arrivée et qui n’ont pas encore développé des signes et symptômes d’infection respiratoire aigüe devront être placés en observation, soit en quarantaine domici- liaire ou en quarantaine institutionnelle. | Always wash your hands with soap and water, or use products made from it. Cough or sneeze into your elbows or into a one-time handkerchief. Remember to always wash your hands before touching your mouth, eyes and nose. Protect yourself in case you have to stay close to or stuck with someone who is breathing hard, coughing or sneezing. Travelers returning from affected areas or who have had contact with a confirmed case within 14 days of arrival and who have not yet developed signs and symptoms of acute respiratory infection should be placed under observation or quarantine residential or institutional quarantine. |
| If you are sick | What to do if you are sick | Not indicated | If you are in Haiti, have traveled to any of the affected areas within the last 14 days and feel sick with fever, cough, or difficulty breathing, Call the Ministry of Public Health’ s center of epidemiology at 4343 3333 |
|  | Threshold to contact healthcare provider | Not indicated | If you are in Haiti, have traveled to any of the affected areas within the last 14 days and feel sick with fever, cough, or difficulty breathing, Call the Ministry of Public Health’ s center of epidemiology at 4343 3333. There is no specifc information about contacting your health care provider because most of the population don't have access to healthcare, so most don't have providers to contact. |
|  | Transport to healthcare facilities | Le transfert du cas COVID-19 en isolement institutionnel se fera au moyen d’une am- bulance médicalisée du CAN | The transfer of the COVID-19 case to institutional isolation will be done by a medical ambulance from CAN (National ambulance center) |
